# Surface marker profiling of SH-SY5Y cells enables small molecule screens identifying BMP4 as a modulator of neuroblastoma differentiation

**DOI:** 10.1038/s41598-017-13497-8

**Published:** 2017-10-19

**Authors:** Fraua Christina Ferlemann, Vishal Menon, Alexandra Larisa Condurat, Jochen Rößler, Jan Pruszak

**Affiliations:** 1 Emmy-Noether-Group for Stem Cell Biology, Department of Molecular Embryology, Institute of Anatomy and Cell Biology, Faculty of Medicine, University of Freiburg, Freiburg, Germany; 2 MOTI-VATE Graduate School, Faculty of Medicine, University of Freiburg, Freiburg, Germany; 3 Spemann Graduate School of Biology and Medicine and Faculty of Biology, University of Freiburg, Freiburg, Germany; 4 Department of Pediatric Hematology and Oncology, Medical Center – University of Freiburg, Faculty of Medicine, University of Freiburg, Freiburg, Germany; 5 Institute for Transfusion Medicine and Gene Therapy, Medical Center – University of Freiburg, Faculty of Medicine, University of Freiburg, Freiburg, Germany; 6 Center for Biological Signaling Studies (BIOSS), University of Freiburg, Freiburg, Germany

## Abstract

Neuroblastoma is the most common extra-cranial solid tumor in children. Its broad spectrum of clinical outcomes reflects the underlying inherent cellular heterogeneity. As current treatments often do not lead to tumor eradication, there is a need to better define therapy-resistant neuroblastoma and to identify new modulatory molecules. To this end, we performed the first comprehensive flow cytometric characterization of surface molecule expression in neuroblastoma cell lines. Exploiting an established clustering algorithm (SPADE) for unbiased visualization of cellular subsets, we conducted a multiwell screen for small molecule modulators of neuroblastoma phenotype. In addition to SH-SY5Y cells, the SH-EP, BE(2)-M17 and Kelly lines were included in follow-up analysis as *in vitro* models of neuroblastoma. A combinatorial detection of glycoprotein epitopes (CD15, CD24, CD44, CD57, TrkA) and the chemokine receptor CXCR4 (CD184) enabled the quantitative identification of SPADE-defined clusters differentially responding to small molecules. Exposure to bone morphogenetic protein (BMP)-4 was found to enhance a TrkA^high^/CD15^−^/CD184^−^ neuroblastoma cellular subset, accompanied by a reduction in doublecortin-positive neuroblasts and of NMYC protein expression in SH-SY5Y cells. Beyond yielding novel marker candidates for studying neuroblastoma pathology, our approach may provide tools for improved pharmacological screens towards developing novel avenues of neuroblastoma diagnosis and treatment.

## Introduction

Neuroblastoma (NB) is the most common extra-cranial solid tumor in infants and the fourth most common cancer in children. Developing from cells derived from the embryonic neural crest^1^, it exhibits considerable heterogeneity with respect to tumor histology and clinical outcome^2–4^. Depending on localization, dissemination, genetic characteristics and patient age, three risk groups and four distinct stages have most commonly been defined^5^. Tumors defined as Stage 4 are particularly heterogeneous, ranging from spontaneous regression to highly aggressive tumor entities^6^. The five-year event-free survival rate of patients suffering from a high-risk tumor stagnates at 40% to 50%^7^ and overall mortality due to NB and other malignancies of the nervous system remains at 29% of all childhood cancer deaths^8^. Besides tumor imaging using computed tomography (CT) or magnetic resonance imaging (MRI) and the detection of urine catecholamine metabolites, biopsies of tumor tissue are required for risk-group assignment and subsequent treatment stratification. Histological features including stroma content, grade of differentiation and the so-called Shimada mitosis-karyorrhexis index serve as important prognostic variables. Common immunohistochemical markers for NB primary tumors and metastases include synaptophysin and the transcription factor PHOX2B, however, with limited specificity^9^. Also, electron microscopic detection of neurosecretory granules and fluorescence *in situ* hybridization (FISH) of the proto-oncogene *NMYC* have been applied in attempts to further differentiate NB biopsy material^2,10^. Genetically, amplification of *NMYC* and expression of the resulting protein, DNA ploidy as well as segmental aberrations of chromosome 11q are used to predict disease outcome^11^.

Depending on the risk-group, current treatment options for NB range from observation to a combination of chemotherapy, surgery, radiation therapy, myeloablative therapy and stem cell transplantation, as well as treatment with isotretinoin (13-cis retinoic acid (RA)), and immunotherapy^5^. The use of 13-cis-RA has been found to improve the survival of children affected by Stage 4 NB by either promoting neuronal differentiation or an apoptotic fate. However, RA is ineffective in some patients, and the underlying mechanisms for selective RA responsiveness remain elusive^12^. Despite many previous studies which have focused on morphological and biochemical differences within NB cells, the cellular heterogeneity of NB has not been resolved in detail^13,14^. While transgenic, syngeneic or xenograft mouse models represent clinically relevant tools for studying NB growth and metastasis^15–18^, cell-based models are the system of choice to determine tumor cell characteristics and to identify pharmacological candidates and assess their efficacy^19,20^. In NB *in vitro* models, commonly three different cell types have been distinguished on a morphological basis: “N-type” showing properties of noradrenergic neurons, “S-type” (substrate-adherent) as a mesenchymal subset showing fibronectin and vimentin expression and the intermediate “I-type” with a mixed expression pattern^21^. These morphologically distinguishable cell types also differ regarding their behavior: N-type cells have been shown to be malignant, whereas S-type cells have been reported to bear reduced malignancy risk, and the stem cell-like I-type cells exhibit the highest malignancy potential of all three^22^. Also, specific phenotypes of NB cells have been linked to the expression of distinct surface molecules. The neurotrophin receptors TrkA and TrkB have been established as prognostic tools of biologically favorable versus biologically unfavorable NB, respectively^23^. Moreover, responsiveness to all-trans RA treatment has been associated with high expression levels of TrkA^24^. Combaret *et al*. correlated the expression of the hyaluronic acid receptor CD (cluster of differentiation) 44 and absence of *NMYC* amplification with a more favorable outcome of NB^25^. Besides, cells with tumor initiation capacity often lack CD44 expression whilst being positive for CD24, a marker for high-risk NB tumors^26,27^. The expression of various integrin subunits seems to be down-regulated in *NMYC*-amplified NB cells^28^. Schlitter *et al*. correlated a high expression of CD57 with an undifferentiated phenotype and aggressive behavior^29^. Furthermore, the disialoganglioside GD2, expressed on neuroblastic cells and mature neurons, is not only of use to identify NB cell populations co-expressing CD56 and CD81^30^, but also plays an important role for modern immune therapy approaches^1^. Notably, however, single markers may not suffice to fully resolve the cellular heterogeneity underlying the differences in tumor behavior and responsiveness to treatment. As it is known from other fields of cancer research, exhaustive surfaceome characterization and the high-dimensional investigation of combinatorial expression patterns can be used to further classify tumor entities as well as to follow-up on treatment results^31,32^. Beyond its established role in hematology and immunology, flow cytometry has evolved into a method widely used in other contexts to define a target population with a suitable combination of surface antigens^33,34^. In the field of neural stem cells and neural tumor stem cells, flow cytometry has been used to further resolve tumor heterogeneity via surface antigen expression analyses^35,36^. Moreover, as described by Ferreira-Facio *et al*., multiparameter flow cytometry can be a valuable tool for diagnostic screening and classification of pediatric cancer entities^30^. The analysis of such multidimensional flow cytometric data remains often subjective to the investigator’s interpretation as the user has to “gate” putative subpopulations for downstream analysis. Manual gating strategies rely on the investigator’s knowledge, interests and previous assumptions about subpopulations and are susceptible to variations across researchers^37^. For a more objective interpretation and a better visualization of multidimensional flow cytometry data, a range of tools have recently been developed^38^. Here, we used an established density-based algorithm (spanning-tree progression analysis of density-normalized events; SPADE) which clusters data depending on similar expression patterns and allows color-coded visualization of median intensities of markers^39^. Overall, we aim to provide a broad characterization of surface antigen profiles associated with NB cellular subtypes. In addition, we apply the resulting marker codes in multiwell-based screens of small molecule candidates to modulate NB subpopulations and to identify the signaling pathways engaged.

## Results

### A surface antigen expression profile of neuroblastoma cells

**Figure 1:**
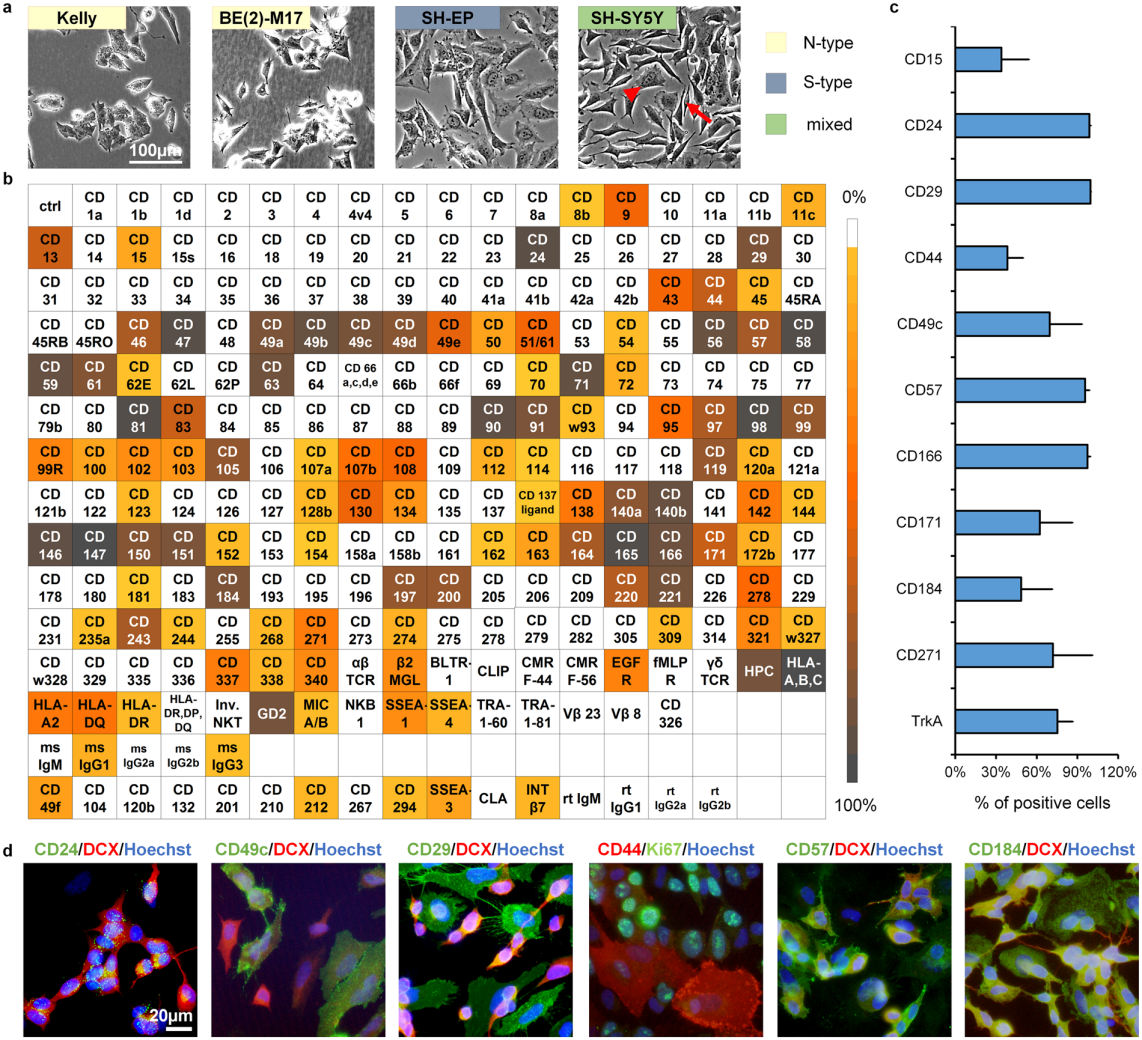
SH-SY5Y cells as a model for the heterogeneity of NB. (**a**) Phase contrast images displaying the heterogeneity of NB cells within and across different cell lines as indicated (scale bar = 100 µm;  indicates an N-type,  an S-type NB cell present in the SH-SY5Y cell line, right panel). (**b**) Flow cytometric screen of the expression levels of 242 surface molecules on SH-SY5Y cells. The heat map graph illustrates the percentage of positive cells for specific antigens within the overall live cell population (color scale; n = 1). Boxes shaded in white represent a ≤ 5% cut-off threshold value. CD: cluster of differentiation. (**c**) Expression of surface marker candidates selected for subsequent analysis under serum-free conditions (n ≥ 3; error bars represent standard deviation). (**d**) Immunofluorescence analysis confirming surface marker expression patterns and cellular heterogeneity of the SH-SY5Y NB line (scale bar = 20 µm).

Established primary tumor- and metastasis-derived NB cell lines display distinct morphologies that reflect their heterogeneous genetic make-up. For instance, the Kelly and BE(2)-M17 cell lines bearing an *NMYC*-amplification are associated with NB aggressiveness (N-type). In contrast, the SH-EP cell line, subcloned from SK-N-SH^21^ lacks *NMYC*-amplification and displays S-type morphology (Fig. 1a). An *in vitro* model comprising both cell types is the SH-SY5Y line, also subcloned from SK-N-SH. Pursuing a first comprehensive analysis of surface antigens expressed in NB, we determined the expression levels of 242 surface antigens on SH-SY5Y cells (Fig. 1b). Surface antigens important for cell adhesion (including CD9, CD24, CD57, CD63, CD81, CD146, CD151)^26,27,29,40–42^ and members of the integrin family (CD29, CD49a to CD49e, CD61, CD98) were highly expressed on SH-SY5Y cells (Table 1). Surface proteins and receptors involved in immune response and immunomodulation were also highly present on SH-SY5Y cells (CD46, CD47, CD59, CD197, CD200)^43–46^. Other highly expressed markers play roles in nutrient uptake (CD71, CD220)^47,48^, detection of mitogenic as well as apoptotic signals (CD140A, CD140B, CD221)^49^, migration of neural crest cells during embryological development (CD184)^50–53^ or are strongly associated with tumor invasion (CD56, CD97, CD146, CD147, CD166)^41,54,55^. Also, factors critical for normal neurohistogenesis (CD171)^26,56^, metastasis of NB (CD44)^27,57^ and modulation of CD184 expression (CD13)^58^ were identified with expression levels >50% on the NB cells. A subset of the identified markers was chosen for further analysis based on their established and/or putative roles in neural stemness (CD15, CD29), neural crest development (CD57, CD271), migration (CD49c, CD49e, CD166) or NB (CD24, CD44, CD184) and for their capacity to identify additional cellular subsets when applied in bivariate combinations in flow cytometric assays (CD166, CD171, CD230). In addition, TrkA was included as an established marker favorably associated with NB prognosis^23^ (Fig. 1c and Supplementary Figure S1). To facilitate standardization and reproducibility of the subsequent small molecule screens, serum-free conditions were chosen, yet overall similar expression levels were observed when compared to standard proliferation conditions (Supplementary Figure S2). Merely CD271 exhibited a higher expression level than expected from the high-throughput profiling (see Fig. 1c) and CD15 as well as CD44 expression levels were found to change depending on the media conditions applied, underlining the context dependencies of such phenotypic analyses (see Supplementary Figure S2). The validity of these markers for identification of cell subsets was confirmed by immunocytochemical (ICC) analysis, where differential expression within NB cultures was observed (Fig. 1d). CD24, a well-known N-type marker, was highly expressed on DCX-positive SH-SY5Y cells (Fig. 1d). In contrast, the integrins CD29 and CD49c as well as CD44 were predominantly associated with DCX-negative S-type cells^35^. CD57 and CD184 were expressed on both cell types to varying degrees (Fig. 1d), and when applied in bivariate combination with CD44 and CD15, respectively, were found to further resolve the existing subpopulations (see Supplementary Figure S1).

**Table 1:** Physiological function and role in cancer of surface antigens highly expressed on SH-SY5Y cells.

	Surface antigen	% positive	Function	Role in cancer	Reference
**Associated with malignant NB**	CD24	>90%	neurite outgrowth, neural migration and neurogenesis	associated with tumor initiation, invasion, proliferation, metastasis	26,27
	CD140b		cell proliferation, chemotaxis, matrix production	mitogen, cellular transformation, malignancy and migration also in NB	49
	CD140a	>80%			
	CD184		guidance of neural crest migration	pro-metastatic in NB	50,52,53
	CD221		cell growth and survival	proliferation in NB	49
	CD57	>70%	marker of early migrating neural crest	high-risk NB	29,40
	CD243		protecting cells against oxidative stress	treatment resistance; NMYC-mediated regulation of MRP1 gene in NB	74
	GD2		oncofetal differentiation agent	aim of immunotherapy with anti-GD2 MAb and tumor-selective delivery of radioisotopes, liposomes, nanoparticles	1
	CD44	>50%	cell-cell and cell-matrix interactions, cell migration	CD44- as NB initiating; metastatic NB if CD44+	27,57
	CD171		nervous system development	EMT, cell migration, malignancy marker in NB	26,56
**Tumor migration, invasion and metastasis**	CD56	>90%	tissue morphogenesis and maintenance of multicellular structure; signal transduction	associated with metastatic progressive cancers with increased motility, migration and invasion abilities	41
	CD166				
	CD146		marker for bone marrow mesenchymal stromal/stem cells		
	CD147		intercellular recognition; stimulates secretion of matrix metalloproteinases		55
	CD97	>70%	adhesion, migration, polarity		54
	CD13	>50%	downregulation of CD184 and modulation of SDF1a-induced cell migration		58
**Immuno modulatory effects**	CD15	<25%	known stem cell marker involved in cell adhesion	involved in cell proliferation and tumor metastasis as well as tumor initiation; correlated with immune system evasion	26,60,75
	CD47	>90%	self-recognition	immunological evasion, regulation of cancer cell invasion and metastasis, cancer recurrence, expressed on cancer stem cells	44
	CD59		inhibitor of the complement system; regulation of T-cell activation	tumor cell resistance to antibody-based therapy by preventing complement cascade	43
	HLA-A,-B,-C	>80%	T-cell-mediated immune surveillance	escape mechanism if downregulated	76
	CD46	>70%	protection against damage from the complement system	tumor cell resistance to antibody-based therapy by preventing complement cascade	43
	CD197		T cell and dendritic cell migration to initiate acquired immune response	tumor progression and metastasis	45
	CD200		peripheral immune tolerance and regulation	immune evasion and tumor escape	46
**Tetraspanins**	CD81	>90%	interaction with receptors and signaling molecules; participation in adhesion, migration, apoptosis	broad effects on cancer; expression might be correlated with prognosis	42
	CD63	>80%			
	CD151				
	CD9	>50%			
**Integrin signaling**	CD98	>90%	cell survival, proliferation, adhesion and migration	enhanced integrin signaling	77
	CD49b			functions in cancer, such as in controlling cell survival, facilitating metastasis	78
	CD29	>80%			
	CD49a				
	CD49c				
	CD49d				
	CD61				
	CD49e	>50%			
	CD51/61				
**Nutrition uptake**	CD71	>90%	iron uptake, regulation of cell growth	upregulated in metastatic and drug resistant tumors	48
	CD220	>70%	insulin receptor; uptake of amino-acids, cell survival, migration	regulation of cell growth in cancer	47
**Associated with biologically favorable NB**	TrkA	>70%	signal transduction of BDNF and NGF as a complex with CD271	prognostic factor for NB tumor as *NMYC*-amplification is inversely correlated with TrkA-positivity	23,79,80
	CD271	<40%	receptor for NGF, NT-3 or BDNF depending on the co-receptor	depending on the co-expression with TrkA or TrkB, CD271 can be found on malignant and biological favorable NB	81,82

### Combinatorial surface marker analysis resolves neuroblastoma heterogeneity

**Figure 2:**
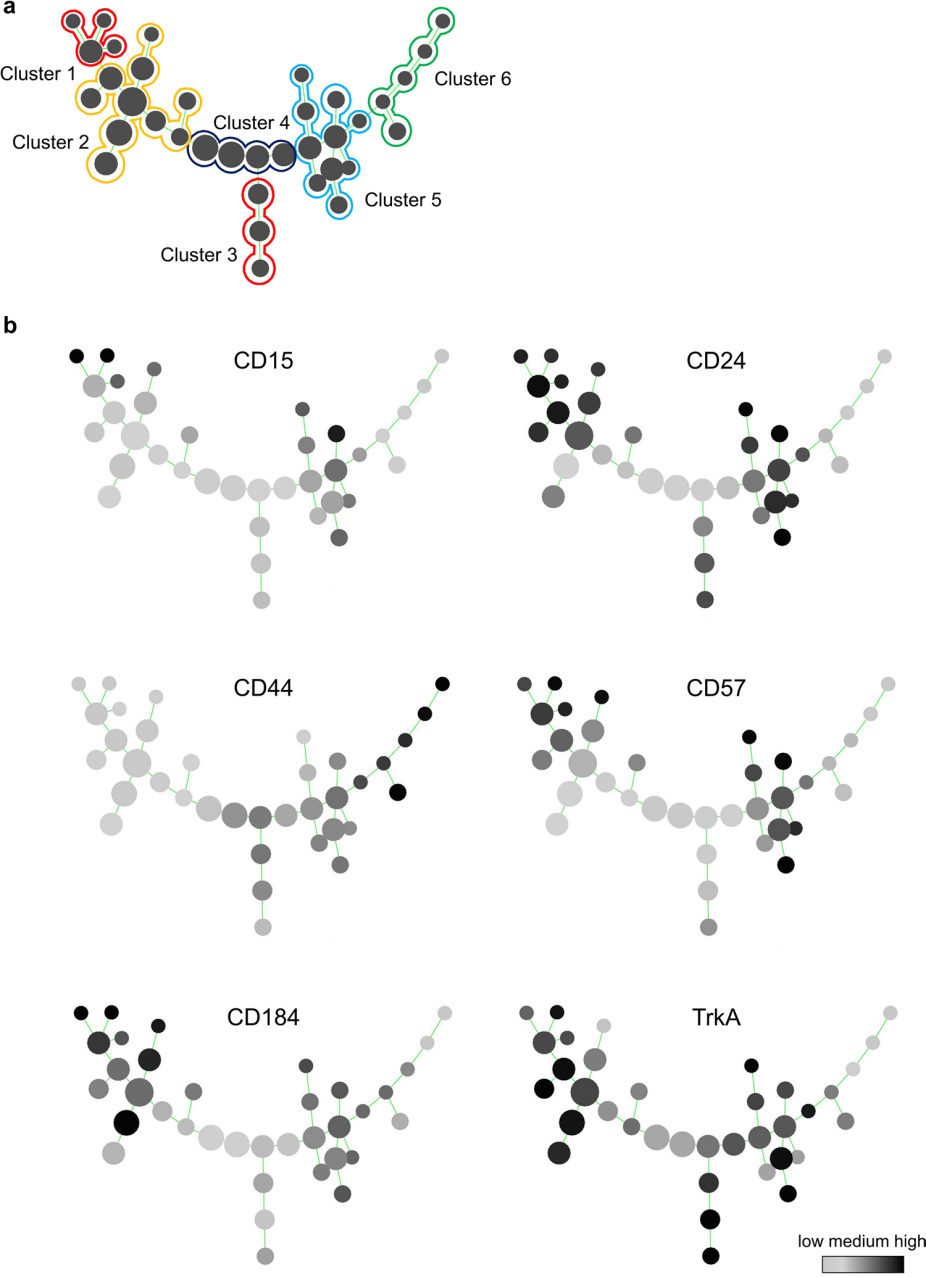
Definition of NB subpopulations via combinatorial surface antigen expression. (**a**) Computational algorithm-based SPADE clustering after multiparametric expression analysis of six combinatorial markers (CD15, CD24, CD44, CD57, CD184, TrkA) to define subpopulations within the SH-SY5Y cell line. Cluster 1 can be characterized as CD15^high^/CD24^high^/CD57^high^/CD184^high^. Cluster 2 displays CD15^−^/CD57^low^/CD184^low^/TrkA^+^ expression. Cluster 3 represents a CD15^−^/CD184^−^ double-negative subpopulation with high TrkA expression. Cluster 4 exhibits little to no expression of the markers included. Cluster 5 shows high levels of CD24, CD57 and TrkA, while Cluster 6 entails the only highly CD44^+^ subset. (**b**) Detailed surface marker expression patterns are shown by grayscale-coded SPADE trees (grayscale code represents relative expression as shown in the reference bar, lower right corner; n ≥ 3).

Hematological and immunological routines exemplify the utility of applying combinatorial labeling strategies based on multiple, not single markers, to resolve cellular heterogeneity in development and malignancy^59^. To minimize investigator bias we subjected the identified markers to combinatorial analysis using the SPADE computational clustering tool. By determining the degree of similarity in combinatorial expression levels of CD15, CD24, CD44, CD57, CD184 and TrkA, six distinct clusters comprising 36 nodes were defined within the SH-SY5Y cell line, thereby considerably extending the resolution beyond the three previously defined subsets (Fig. 2a). We hypothesized that this detailed multifactorial analysis would allow for the detection of subtle shifts of NB phenotypes in response to the application of candidate disease-modifying molecules. Thus, a six marker-based, objectifiable definition of NB cell types was established. High levels of CD44 expression, a marker commonly found within the S-type NB subpopulation, were characteristic for Cluster 6. Cluster 5 exhibited CD15, CD24, CD57, CD184 and TrkA expression, yet none of these markers alone was exclusive for this branch. Cluster 4 showed little to no expression of any of the included markers. Cluster 3 could clearly and uniquely be defined as CD15^−^/CD57^−^/CD184^−^/TrkA^high^. Cluster 2 showed expression of CD24, CD57, CD184 and TrkA, similar to Cluster 5. Notably, Cluster 1 was the only subgroup within the overall population showing a high expression of CD15 in combination with positivity for CD24, CD57 as well as CD184 (Fig. 2a,b). Cell sorting for the CD15^−^/CD44^+^/TrkA^−^, CD15^−^/CD44^−^/TrkA^+^ and CD15^+^/CD44^−^/TrkA^−^subpopulations was conducted to further characterize the clusters 6, 3 and 1, respectively, representing the major branches of the spanning tree. In line with previous reports^60^, cells within Cluster 6 showed S-type morphology and high expression of the migration- and metastasis-associated Hippo pathway effector YAP (Yes-associated protein; YAP1) as well as CD44, while being mostly negative for DCX. Interestingly, based on mere morphological and immunocytochemical analyses, no overt differences between Cluster 1 and Cluster 3 were discernible (Supplementary Figures S5,S6 and S7), illustrating the utility of combinatorial flow cytometric screens to resolve cellular complexity and to identify the clearly differential responsiveness of NB subsets (see Fig. 2).

### A multiwell platform for small molecule screens

**Figure 3:**
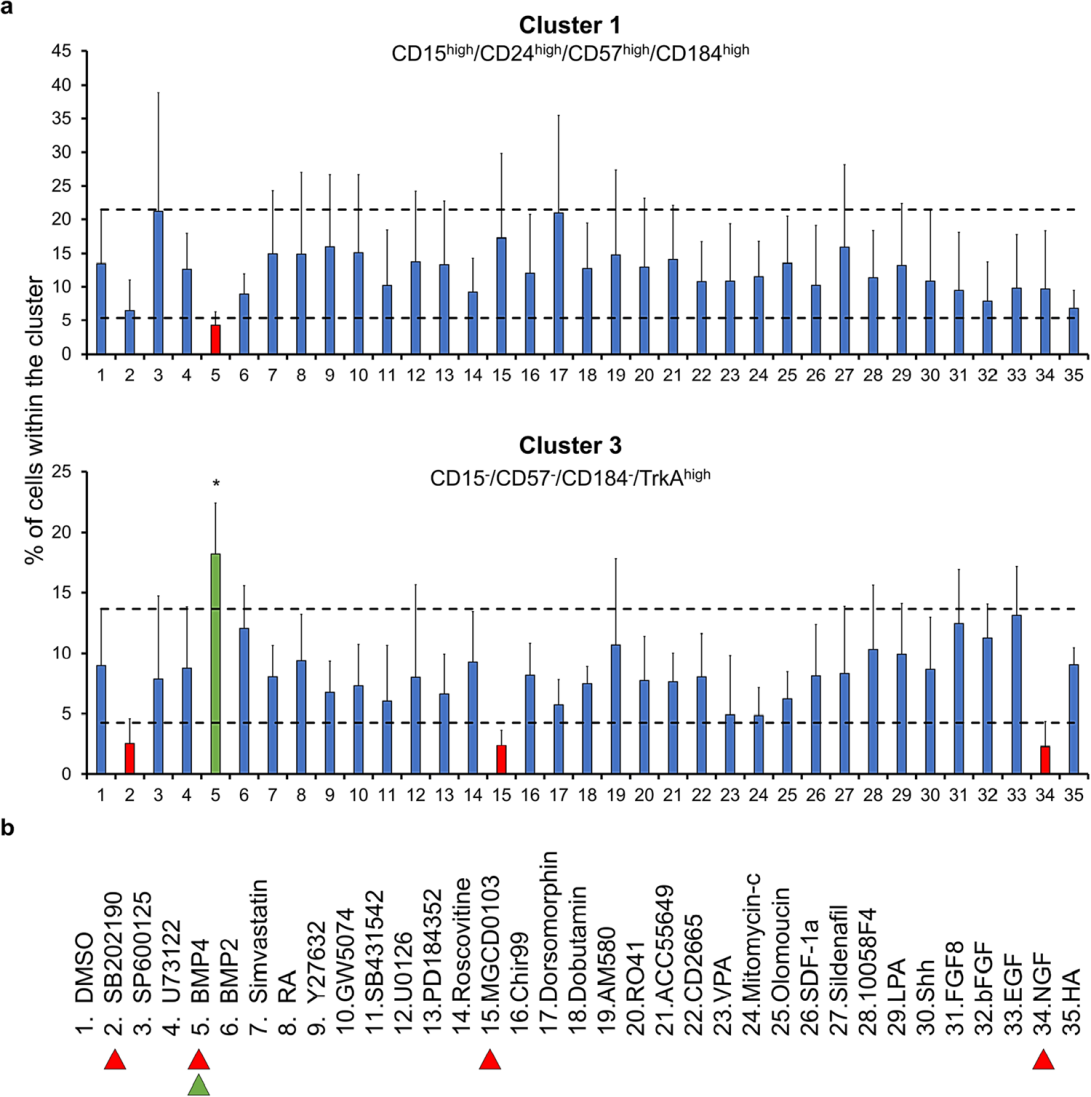
Detecting distinctive responses of NB subpopulations to specific small molecule modulators. (**a**) Flow cytometry analysis of SPADE-defined NB subpopulation responsiveness to small molecule modulators following 48 hours of treatment (concentrations as indicated in Supplementary Table S2). In the small molecule screen conducted, four reagents revealed the capacity for enhancing or decreasing a specific subpopulation. Specifically, BMP4 was able to enhance neuronal differentiation whilst decreasing the amount of CD15^high^/CD24^high^/CD57^high^/CD184^high^ cells (Cluster 1). Error bars represent standard deviation. Dashed lines represent 1 standard deviation from DMSO control. *p ≤ 0.05 in a one-way ANOVA test followed by Dunnett’s multiple comparisons test comparing all columns to the DMSO control. (**b**) List of small molecules applied in the screen. Colored arrowheads highlight  up-, or  downregulation of subpopulations induced by the respective molecules (n ≥ 3).

As a proof-of-concept, we conducted a low-throughput screen for representatives of NB-associated signaling pathways: four hits from the screening panel of small molecules were identified by their capacity to either enhance or decrease particular SPADE-defined clusters beyond one standard deviation of the mean control values (Fig. 3, Supplementary Figures S3 and S4). Among the small molecules tested, the morphogenetic protein BMP4 was the only one to show a significant effect on any of the clusters. BMP4 was able to decrease the CD15^high^/CD24^high^/CD57^high^/CD184^high^ marker expression associated with Cluster 1, and to enhance expression of the CD15^−^/CD57^−^/CD184^−^/TrkA^high^ profile characteristic of Cluster 3. Conversely, the p38 MAP kinase inhibitor SB202190, the HDAC inhibitor MGCD0103 as well as NGF, a growth factor capable of maintaining cells in an undifferentiated state, showed the tendency to decrease the relative cellular frequency of this latter subset without reaching statistical significance (Fig. 3a). In turn, the CD15^−^/CD57^−^/CD184^−^/TrkA^high^ (Cluster 3) NB subpopulation might represent a more differentiated and less aggressive type. The only small molecule analyzed in our screen which specifically enhanced this subpopulation was BMP4 (Fig. 3b).

### BMP4 promotes TrkA expression and NB differentiation

**Figure 4:**
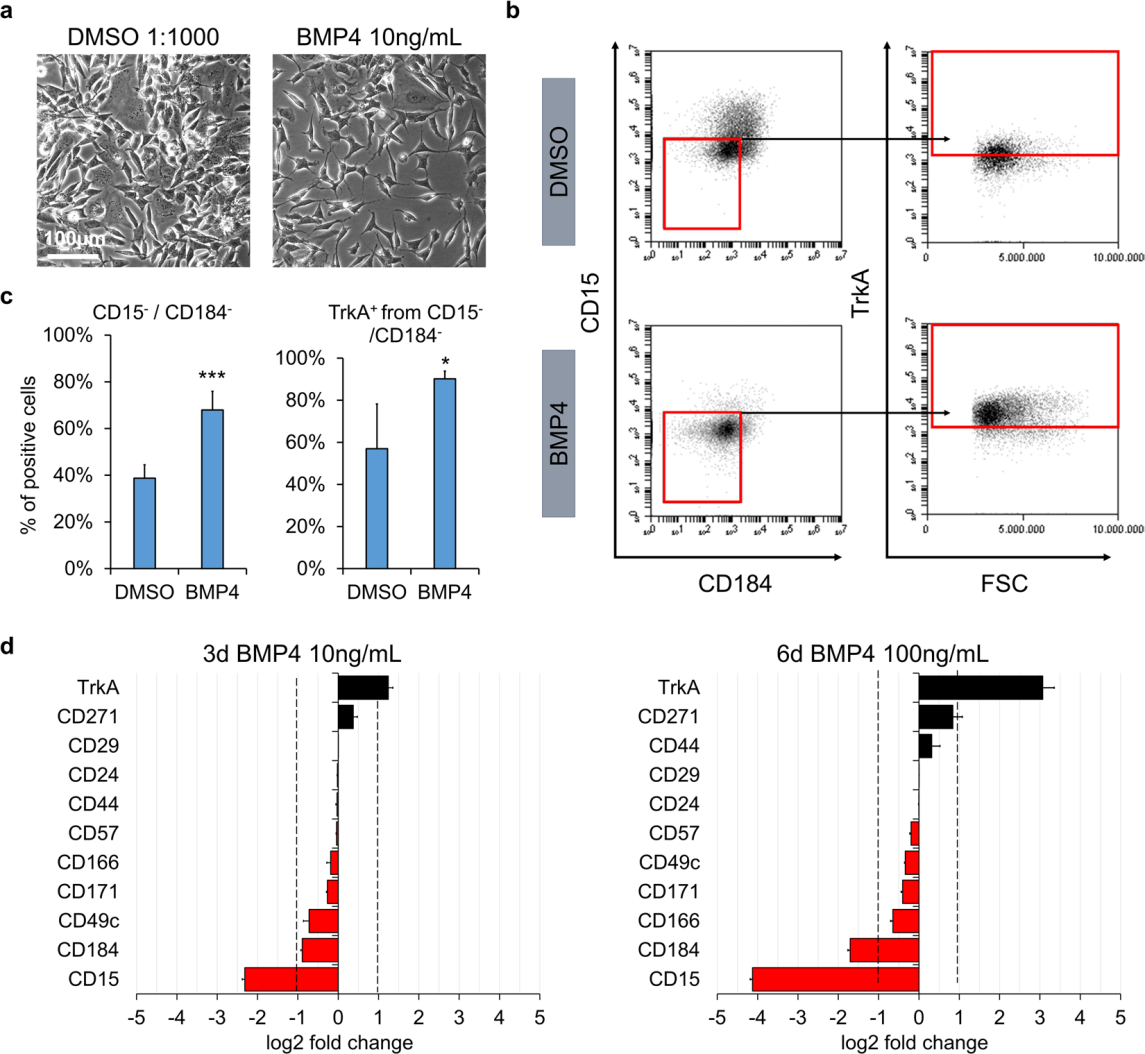
BMP4 enhances a TrkA^+^/CD15^−^/CD184^−^ subpopulation in a dose-dependent manner. **(a)** Phase contrast images illustrating morphological changes of SH-SY5Y cells after 3 days of BMP4 treatment (10 ng/mL) with enhancement of neurite-like extensions. Scale bar = 100 µm. **(b**,**c)** Representative flow cytometric plots and corresponding bar graphs focusing on the surrogate marker candidates TrkA, CD15 and CD184 after 3 days of treatment with 10ng/mL BMP4 (error bars indicate SD; **p ≤ 0.01; ***p ≤ 0.001 in an unpaired Student’s t-test; n ≥ 3) **(d)** Bar graphs represent log2 fold change in the expression pattern of different surface antigens after BMP4 treatment at a concentration of 10 ng/mL for 3 days as well as 100 ng/mL for 6 days. Correlated with increased BMP4 exposure, the most prominent changes occurred with respect to the TrkA, CD184 and CD15 markers (n ≥ 3; dashed lines represent cut-off of 1; red bars represent down-, black bars upregulation; error bars represent standard error of the mean incl. error propagation).

**Figure 5:**
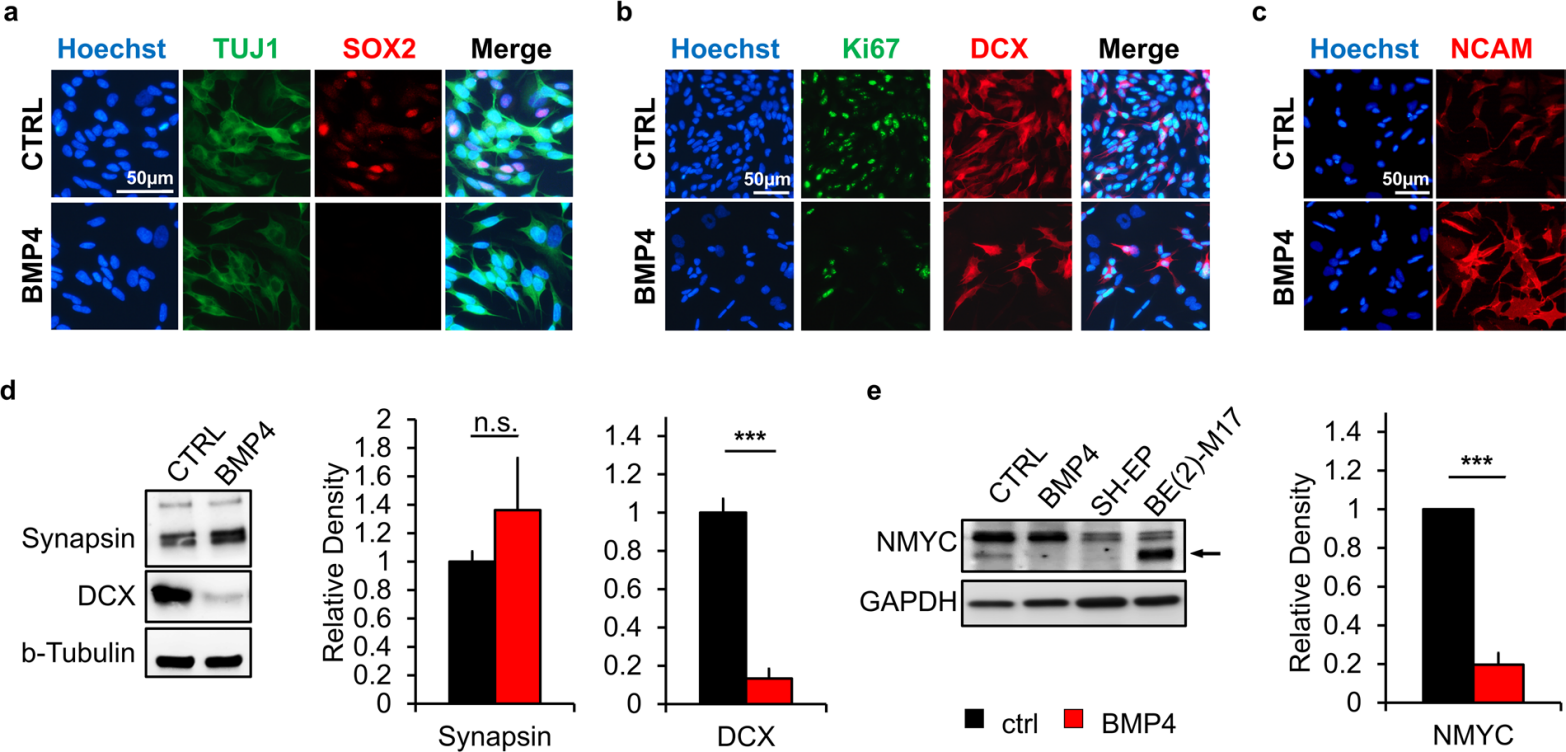
BMP4 promotes NB differentiation. (**a**–**b**) Compared to control conditions, immunofluorescence analysis of BMP4-treated SH-SY5Y cells showed a decrease of stemness-associated SOX2, the proliferation marker Ki-67 and the neuroblast marker DCX. **(c)** In contrast, an increase of neuronal differentiation marker NCAM was observed upon BMP4 treatment. (**d**) Western blot analysis showed maintained to mildly increased synapsin levels and clearly decreased DCX protein content in BE(2)-M17 NB cells (n ≥ 3; n.s. not significant, ***p ≤ 0.001). (**e**) In SH-SY5Y cells, treatment of BMP4 also significantly decreased NMYC content (n ≥ 3; ***p ≤ 0.001).

**Figure 6:**
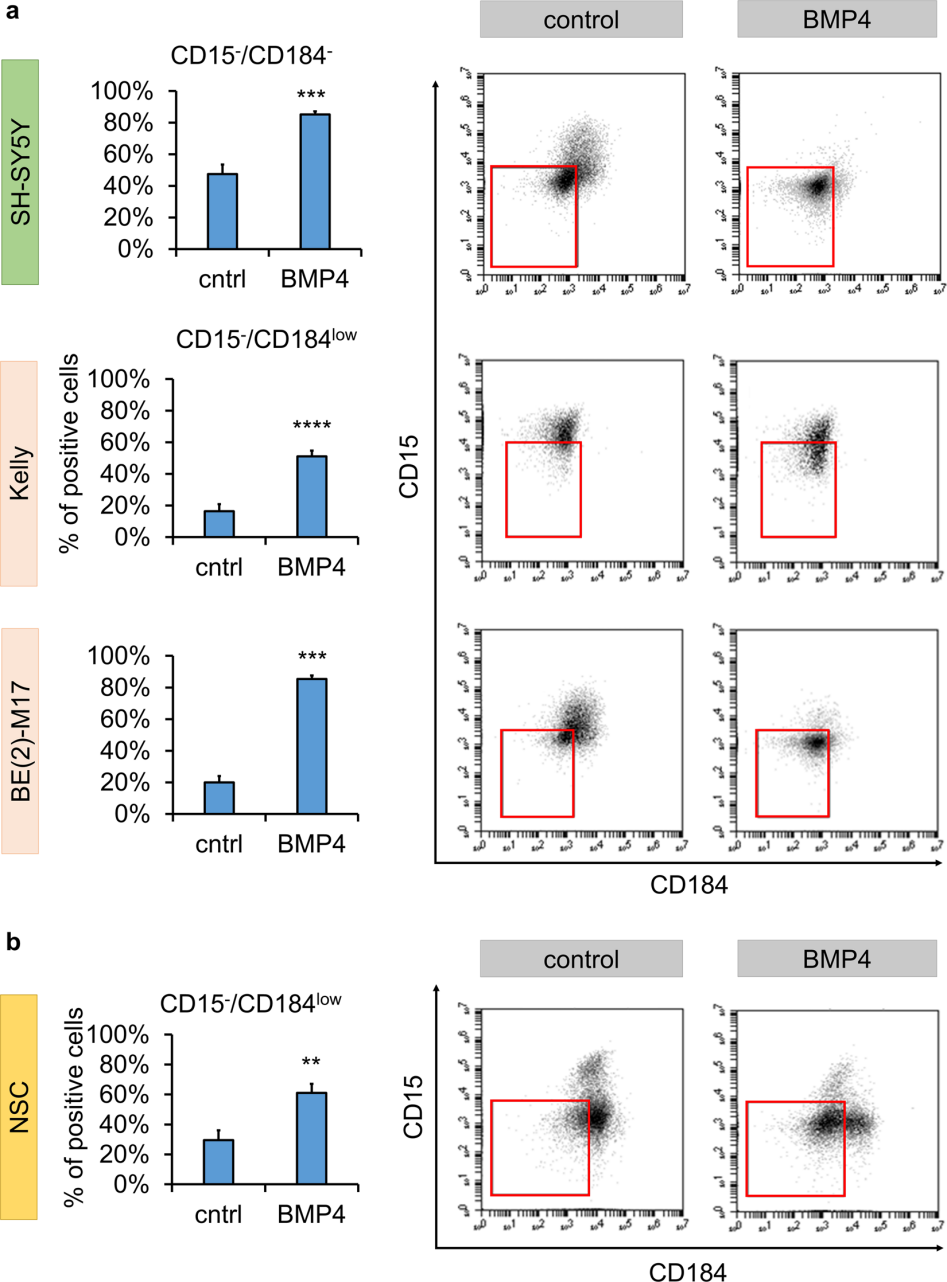
The effect of BMP4 is maintained across different NB and other neural cell lines. (**a**) Quantification and representative dot plots of the CD15^−^/CD184^−^ subpopulation in SH-SY5Y cells and the comparable CD15^−^/CD184^low^ subset in N-type, *NMYC*-amplified Kelly and BE(2)-M17 NB cell lines following 6 days of BMP4 treatment at a concentration of 100 ng/mL. (**b**) Similar effects on diminishing CD15 and CD184 surface antigen expression were also seen in non-transformed human embryonic stem cell derived neural stem cells (NSC) following treatment with 100ng/mL BMP4 for 3 days. Error bars indicate SD; **p ≤ 0.01, ***p ≤ 0.001, ****p ≤ 0.0001, unpaired Student’s t-test; n ≥ 3.

To follow-up on results obtained from the small molecule screen, we analyzed cells treated by the reagents that had shown an effect on the most responsive NB subpopulation clusters. Analogous to the flow cytometric responsiveness, the identified molecules induced phenotypic changes as determined by classic phase contrast analysis, underlining the utility of our flow cytometric paradigm as a surrogate for conventional pharmacological screens by microscopic readout (Supplementary Figure S8). On the other hand, morphological changes induced by BMP4 were relatively subtle. This prompted us to further investigate this effect, which likely would have escaped conventional, non-flow cytometry-based screening paradigms (Fig. 4a). The pattern changes identified could be narrowed down to be largely due to surface expression changes in CD15, CD184 and TrkA (Fig. 4b,c). However, also the expression of the low affinity NGF receptor CD271 (p75) was altered by BMP4 treatment (Supplementary Figure S9). The observed effects proved to be dependent on dosage as well as on duration of treatment (Fig. 4d). Compared to BMP2 and TGFβ-1 (also mediated via the same or similar receptors, respectively) BMP4 exhibited a greater impact with respect to surface marker expression changes when applied at a comparable dose (Supplementary Figure S10). RA, further analyzed due to its current use in NB treatment, was less efficient in the up- and downregulation of TrkA and CD184, respectively, and actually resulted in the upregulation of CD15 (Supplementary Figure S11). The identified effect of BMP4 on the identified CD15^−^/CD184^−^ subpopulation remained consistent across different combinatorial treatments with RA (Supplementary Figure S12) as well as in 15% FBS-containing medium (Supplementary Figure S13). To further investigate the influence of BMP4 on NB phenotype, we analyzed treated NB cells via immunocytochemical and western blot readout. Immunofluorescence revealed that the stemness-associated marker SOX2, the proliferation marker Ki-67 and neuroblast marker DCX were decreased upon BMP4 treatment of SH-SY5Y cells (Fig. 5a,b). Complementing the observed enhancement of neurite extensions (see Fig. 4a), immunoreactivity for the neuronal differentiation marker NCAM was found to be increased (Fig. 5c). In the highly *NMYC*-amplified N-type NB cell line BE(2)-M17, the protein content of DCX was significantly reduced upon exposure to BMP4 while the mature neuronal markers synapsin and MAP2 showed a mild upward trend (Fig. 5d, Supplementary Figure S14). In SH-SY5Y cells, also NMYC protein, a surrogate marker for NB aggressiveness, showed a significant reduction of expression after BMP4 treatment (Fig. 5e). While this was not seen in the highly *NMYC*-amplified BE(2)-M17 cell line when applying the same regimen (10 ng/mL over 3 days), the surface marker changes induced by BMP4 treatment could be somewhat mimicked by siRNA-mediated knockdown of *NMYC* (see Supplementary Figure S14 **)**. Overall, our data indicate that BMP4 may modulate and promote the differentiation of NB cells. Moreover, following up on the potential link to the NMYC network, we could show that the surface marker changes induced by BMP4 could be confirmed on *NMYC*-amplified Kelly and BE(2)-M17 NB lines to a similar degree as seen in non-*NMYC*-amplified SH-SY5Y cells (Fig. 6a). As also non-transformed neural stem cells derived from human embryonic stem cells showed an upregulation of the CD15^−^/CD184^low^ subpopulation upon treatment, the resulting phenotypic changes described herein might represent a generalizable effect of BMP4 signal transduction (Fig. 6b).

## Discussion

Intratumor heterogeneity has been shown to have a major impact on treatment response and clinical outcome in cancer patients^3,4,13,61^. To further resolve the cellular heterogeneity of NB, we performed flow cytometric surface molecule profiling of the widely-used SH-SY5Y NB cell line. The initial comprehensive analysis of overall CD surface marker expression (see Fig. 1, see Table 1) revealed high expression levels of molecules generally involved in tumor invasiveness (CD29, CD44, CD57, CD184) and cell survival (integrins, CD220, CD221) as well as the presence of cancer stem cell-associated markers (CD15, CD24, CD44). We subsequently established a novel subgrouping of NB cells based on the co-expression of specific CD molecules in distinct cell clusters using the SPADE software, an unbiased expression density-based algorithm^39^, focusing on representative markers of neural crest cell development and NB progression: CD15, CD24, CD44, CD57, CD184 and TrkA. Based on the combinatorial co-expression of these markers, the SPADE analysis revealed six main cellular clusters, suggesting the presence of more subsets in NB than the classically described S-, N- and I-type^21^. Previous work showed that CD44-expressing cells are negative for DCX, a marker of neural precursor cells^60^, and display a mesenchymal morphology (see Fig. 1d). Therefore Cluster 6, defined as CD15^−^/CD44^high^/CD184^−^/TrkA^−^, was considered to share similarity with the previously defined S-type NB cells proposed by Ross and colleagues^22^. CD44 is a cell surface glycoprotein with a controversial role in the context of NB, several reports supporting the presence of functional CD44 as a good prognostic marker^25^, while in other studies CD44 expression has been associated with a metastatic phenotype^57^. In contrast, cells within Cluster 3, exhibiting a CD15^−^/CD57^−^/CD184^−^/TrkA^high^ surface expression profile, do express DCX and present neuronal extensions, suggesting their alignment with the N-subtype NB classification. Notably, in NB DCX is oftentimes co-expressed with proliferative markers such as Ki-67. Cluster 1 is characterized by a CD15^high^/CD24^high^/CD57^high^/CD184^high^ expression profile. The co-expression of these markers could be of particular interest due to their general engagement in various tumorigenic mechanisms. For example, CD15 is a known neural and cancer stem cell marker, involved in tumor metastasis^62^. CD184 (CXCR4) is a chemokine receptor essential for the guidance of neural crest migration^50^, also reported to play a pro-metastatic role in several tumor entities including NB^52,53^. CD57 (HNK1) is an early neural crest marker expressed on migratory neural crest cells^40^, reported to be expressed at high levels in aggressive NBs^29^. The observed co-expression of these surface antigens associated with migratory precursor markers could hint towards an undifferentiated and/or aggressive NB phenotype similar to the reported I-subtype, yet further detailed analysis would be warranted. The other identified clusters may reflect intermediate or transitory phenotypes or functionally distinct NB cellular entities. In addition to drug responsiveness, it will be interesting to assess their respective potencies for tumor initiation, metastases and interconversion dynamics^4^. By extending the options beyond previous morphological analysis, the newly established marker combinations enable detailed NB phenotypic readout via immunostaining and quantitative flow cytometry.

Following the SH-SY5Y surfaceome analysis, we performed a small molecule screen to determine the sensitivity and responsiveness of the characterized clusters to specific compounds, comprising some already employed as anticancer agents. In the screening panel, molecules targeting oncogenic factors, cell proliferation, promoters of apoptosis and/or differentiation were included. Among the resulting hits, decreasing cell frequencies associated with Cluster 3, were nerve growth factor (NGF), the p38 MAP kinase inhibitor SB202190 and the selective histone deacetylase inhibitor MGCD0103 (Mocetinostat), the latter currently being explored in clinical trials for a variety of cancers. Notably, the morphogen BMP4 was the only one of the molecules tested to enhance the same subpopulation (Cluster 3). In addition, cells within Cluster 1 were exclusively affected by exposure to BMP4, resulting in a decrease of this particular cellular subset. BMP-signaling is well known to play a major role at various stages during neural crest cell differentiation^63^ and has been shown to promote cell death in certain tumors^64^. As illustrated by the longer neurite-like extensions seen in phase contrast microscopy (see Fig. 4, Supplementary Figure S8) and phenotypic analysis of neuroblast and neural differentiation markers (see Fig. 5, Supplementary Figure 14) BMP4 treatment appears to promote neuroblastoma differentiation. The reduction in Cluster 1 with a concomitant gain in Cluster 3 may reflect the pro-differentiating effects of BMP4 in SH-SY5Y cells. Interestingly, BMP4 significantly increased the CD15^−^/CD184^−^ subset and TrkA in the SH-SY5Y line as well as in the *NMYC*-amplified BE(2)-M17 and Kelly cell lines alike (see Fig. 6). CD15 and CD184 are known pro-tumorigenic factors in a variety of tumors, while the presence of TrkA is commonly associated with a good prognosis in NB, making BMP4 a compelling therapeutic molecule candidate. Mechanistically, it remains to be shown, whether the enrichment in the CD15^−^/CD184^−^ fraction is caused by a direct or indirect BMP4-mediated regulation of CD15 and/or CD184 expression or, for instance by a depleting/pro-apoptotic effect targeting this subset. Of note, we observed a previously not described modulatory effect of BMP4 on NMYC expression, highlighting its impact on intracellular networks associated with NB malignancy and aggression^11,15,65^. The observed enhancement of TrkA expression after BMP4 paired with a decrease of NMYC is congruent with the role of TrkA as a marker of non-*NMYC* amplified NB entities. In line with this, direct modulation of *NMYC* levels via siRNA replicated the effects of BMP4 on CD15 and TrkA surface expression. In contrast, RA, a natural morphogen known for its pleiotropic effects in various tissues and an established member of the arsenal to treat high-risk NB, showed no significant effects on either one of the analyzed clusters in the *in vitro* systems investigated here. This may reflect the varied effects of RA in SH-SY5Y cells, with previous reports showing pro-apoptotic and differentiation effects^1,12^ as well as pro-migratory phenotypes^60,66^. We have previously illustrated that the observed pro-migratory RA-induced phenotype is largely associated with activity of the Hippo pathway effectors YAP/TAZ, and requires the joint action of RA and Hippo signaling in regulating a CD15^−^/CD44^+^/CD49d^+^ SH-SY5Y subset^60^. Overall, the single treatments used in the current study led to moderate global effects since only particular clusters were affected, indicating that NB heterogeneity poses a major challenge in terms of efficient drug targeting. Dual stimulation with different molecules in a sequential manner may target multiple clusters and increase overall impact. When applying RA either before or after BMP4 treatment an enhanced effect on TrkA and CD271 upregulation and CD49c downregulation was observed. Interestingly, BMP4 was able to overwrite the observed upregulation of CD15 seen with RA treatment alone (see Supplementary Figures S11 vs. S12). These examples suggest elaborate intracellular signaling modulation that can lead to both additive, but also a putative inhibitory effect of BMP4 on RA signaling reflecting the complex interaction between the two signaling cascades at early and late stages of neural crest development^63,67^. Profound synergism of BMP4 and RA co-stimulation, leading to increased pro-apoptotic effects, have been previously reported in P19 embryonal carcinoma cells^67^ and in retinoblastoma cell lines^64^. This suggests that perhaps a combinatorial, rather than sequential treatment strategy could potentially be more effective in NB cells as well. Combined treatments including RA and histone deacetylase inhibitors^68–70^ or the protease inhibitor MG132^71^ have been shown to have more pronounced and synergistic effects in targeting NB cells. The identification of surface antigens associated with specific tumor subclones will prove useful for enhancing our insight into the dynamics of NB progression, cellular interconversion and potency, which may enable the development of more precise and ultimately more efficient combinatorial treatment paradigms.

In summary, flow cytometric characterization of SH-SY5Y cells provides a first comprehensive overview of NB-associated CD surface molecule antigens, yielding ample options for pathology studies and potential future immunotherapeutic paradigms. The combinatorial detection of glycoprotein epitopes (CD15, CD24, CD44, CD57, TrkA) and the chemokine receptor CXCR4 (CD184) uncovers NB cellular heterogeneity. Moreover, the computational SPADE-clustering approach provides an unbiased means of quantitatively identifying molecules targeting specific NB cellular subsets in a heterogeneous setting in multiwell screens. As a case in point, BMP4 was identified as a compelling candidate molecule promoting NB cell line differentiation, exhibiting the capacity to decrease expression of CD15 and CD184 antigens as well as the neuroblast marker DCX and malignancy-associated NMYC, while simultaneously enhancing TrkA expression.

## Methods

### Cell culture

The human NB cell line SH-SY5Y was obtained from *Deutsche Sammlung von Mikroorganismen und Zellkulturen* (DSMZ). SH-SY5Y cells are N-type cells subcloned from the SK-N-SH NB cell line^21^ which was obtained by bone-marrow aspiration of a 4-year-old female patient with metastasized NB^72^. The cells were cultured in a 1:1 mixture of Dulbecco’s Modified Eagle Medium and Ham’s F12 (Gibco® Life Technologies) (DMEM/F12) supplemented with 15% heat-inactivated fetal bovine serum (FBS; Gibco® Life Technologies) or 10% knock-out serum replacement, when specified, and 1% non-essential amino acids (NEAA; Gibco® Life Technologies). The human NB cell lines Kelly and SH-EP, both obtained from Prof. Dr. Jochen Rößler, *Zentrum für Kinder- und Jugendmedizin*, *Universitätsklinikum Freiburg*, were cultured in Roswell Park Memorial Institute (RPMI) medium (Gibco® Life Technologies) supplemented with 10% heat-inactivated FBS. The human NB cell line BE(2)-M17, obtained from ATCC, was cultured in DMEM/F12 supplemented with 10% heat-inactivated FBS. The neural stem cells (NSC), derived from human embryonic stem cells (H9; WiCell), were cultured in poly-l-ornithine/laminin(P/L)-coated tissue culture flasks in DMEM/F12 medium with 1% N2 supplement (Stemcell Technologies). All cells were cultured in an atmosphere of 5% CO_2_ at 37 °C. For NB cell lines the medium was changed at least three times per week with passaging upon reaching ca. 80% confluency. NSCs received fresh media every day and were subcultured after three days as previously described^60^.

### Small molecule treatment

For the treatment with small molecules, cells were seeded at a density of 2.4 × 10^4^ to 6.25 × 10^4^ cells per cm² (depending on length of treatment for SH-SY5Y cells), 3 × 10^4^ cells per cm² (Kelly and BE(2)-M17) or 1.5 × 10^4^ cells per cm² (SH-EP) and cultured overnight in their standard growth medium, before starting the treatment in medium supplemented with 10% knock-out serum replacement (KO-SR). Treatment occurred for two days without media change and for three days and six days with one media change. See Supplementary Table S2 for a list of small molecules and concentrations used. Growth and morphological changes were monitored under the microscope. After treatment, cells were harvested for flow cytometry analysis.

### Screen

The small molecule screen was conducted in a 96-well-plate (Cellstar®, Greiner Bio-One). SH-SY5Y cells were plated at a density of 5 × 10^4^ cells per cm^2^ and kept in FBS containing medium overnight followed by the treatment as indicated for 48 hours in DMEM/F12 medium supplemented with 10% KO-SR. Cells were harvested using TrypLE and re-suspended in PBS with 2% FBS to obtain a single cell suspension. Surface antigens were labeled as described before^35,73^ by incubating cells with conjugated antibodies (Supplementary Table S1 **)** for 30 min in the dark at room temperature on a shaker. Centrifugation steps were conducted in a refrigerated table microcentrifuge (Preqlab Perfect Spin) at 2000 rpm (376 rcf) for 4 min. After staining, cells were washed three times before flow cytometric data acquisition using a BD LSRFortessa^TM^ equipped with 405 nm, 488 nm, 561 nm and 640 nm lasers. Data was analyzed using the SPADE 3.0 software (Qiu *et al*. 2011; http://pengqiu.gatech.edu/software/SPADE/)^39^ for computers without Matlab. Compensation was taken into account, arcsin factor was set to 150 and the program clustered for 36 populations. Annotations were drawn regarding the expression pattern of FITC, PE, APC and eFLuor460.

### Flow cytometry

Cells were harvested and prepared for data collection as described for the small molecule screen. Centrifugation steps were conducted using two refrigerated table microcentrifuges: Preqlab Perfect Spin 24 R at 2000 rpm (376 rcf) and Eppendorf Centrifuge 5804 R at 1880 rpm (376 rcf) for 4 minutes. The stained cells were analyzed on a BD™ Accuri® C6 benchtop cytometer equipped with FL1 (533/30), FL2 (585/40) and FL4 (675/25) bandpass filters. Samples were run at 66 µL/min flow rate at 22 µm core size. Data were analyzed using BD™ CFlow® Plus software version 1.0.227.4 © 2008. Gates for detecting positive staining were set against unstained controls. A threshold of 0.5% of unstained cells within the positive gate was set. Where appropriate, compensation was applied according to single-stained control samples of the same cell type included in each individual experiment. The compensation values were generated to regain the set threshold of 0.5% in all channels (FL1, FL2, FL4). For very bright stainings, however, higher thresholds up to 1% within the positive gate were allowed after compensation. See Supplementary Table S3 for information on the antibodies used.

### Immunocytochemistry

The cells were cultured in a 24-well-plate at a density of 3 × 10^4^ cells per cm² on gelatin-coated glass cover slips. Some surface antigens were immunolabeled prior to fixation. See Supplementary Table S4 for the mode of staining and concentration of antibodies used. Cells were fixed in 4% PFA for 30 min at room temperature and permeabilized in 0.5% Triton-X100 in PBS for 10 min. 1% BSA and 10% normal donkey (NDS) or normal goat serum (NGS) in PBS was used for 30 min blocking followed by hybridization with the primary antibody in blocking solution overnight at 4 °C. After four washing steps with PBS for 5 min, secondary antibodies were incubated at room temperature for 1 hour. Another four washing steps in PBS for 5 min preceded a short washing in H_2_O, followed by the mounting of the coverslip in 10 µL ProLong® Diamond Antifade Mountant (Life Technologies) on a glass slide. After drying, pictures were generated using the Axioplan-2/Zeiss microscope. For visualization, AxioVision Special Edition 64 Release 4.9.1 was used. Using secondary antibody only controls, true positive staining was adjusted using the Zen 2.3 (blue edition) (© Carl Zeiss Microscopy GmbH).

### Protein Blot Analysis

Cells were treated and harvested as described above. Cells were lysed in sample buffer and total protein concentration was measured using the nanodrop-1000 (Peqlab). 15 µg of protein was loaded and subjected to electrophoretic separation. Wet blotting was performed in 1x transfer buffer with 100 V for 1 hour. Membranes were blocked for 1 hour at room temperature followed by overnight incubation with the primary antibody at 4 °C. The secondary antibody was added after four washing steps for 5 min and incubated for 1 hour at room temperature. Proteins were detected using chemiluminescence. See Supplementary Tables S5 and S6 for information on buffer composition and other products.

### RNA interference

siRNA-mediated knockdown of *NMYC* was conducted in SH-SY5Y cells as well as in *NMYC*-amplified BE(2)-M17 cells as described previously^61^. A combination of three siRNAs targeting *NMYC* (see Supplementary Table S7) was used and a repeat knockdown was conducted after 24 hours to increase efficiency. Scrambled non-specific siRNA served as a negative control.

### Statistics

Flow cytometry data illustrated in heat maps and bar graphs represent the arithmetic means of experimental repeats. For each experiment, a minimum of 5,000 cells were acquired to generate the expression values. At least three biological repeats were conducted for each set of experiments. Error bars indicate standard deviation (SD). Two-tailed Student’s t-test was applied to investigate significant changes of CD marker expression depending on medium condition and treatment. If necessary, multiple testing corrections were applied. Specific information about the applied tests will be found in the according figure legends. For statistical analysis, GraphPad Prism Version 5 and 7 were used.

### Data availability

All data generated or analyzed during this study are included in this published article (and its Supplementary Information files).

## Electronic supplementary material

Supplementary Material
