# Supplementary material for: Surface marker profiling of SH-SY5Y cells enables small molecule screens identifying BMP4 as a modulator of neuroblastoma differentiation
Source: Sci Rep. 2017 Oct 19;7:13612. doi: 10.1038/s41598-017-13497-8 (PMC5648761; doi:10.1038/s41598-017-13497-8)
Supplement: Supplementary file 1 — Supplementary Material [file 41598_2017_13497_MOESM1_ESM.pdf]

SUPPLEMENTARY INFORMATION

Surface marker profiling of SH-SY5Y cells enables small molecule screens identifying BMP4 as a modulator of neuroblastoma differentiation

Fraua Christina Ferlemann[1,2*], Vishal Menon[1,3*], Alexandra Larisa Condurat[1,3], Jochen Rößler[4], Jan Pruszak[1,5,6#]

[1]*Emmy-Noether-Group for Stem Cell Biology, Department of Molecular Embryology, Institute of Anatomy and Cell Biology, Faculty of Medicine, University of Freiburg, Freiburg, Germany*

[2]*MOTI-VATE Graduate School, Faculty of Medicine, University of Freiburg, Freiburg, Germany*

[3]*Spemann Graduate School of Biology and Medicine and Faculty of Biology, University of Freiburg, Freiburg, Germany*

[4]*Department of Pediatric Hematology and Oncology, Medical Center – University of Freiburg, Faculty of Medicine, University of Freiburg, Germany*

[5]*Institute for Transfusion Medicine and Gene Therapy, Medical Center – University of Freiburg, Faculty of Medicine, University of Freiburg, Germany*

[6]*Center for Biological Signaling Studies (BIOSS), University of Freiburg, Freiburg, Germany*

[*]These authors contributed equally.

[#]To whom correspondence should be addressed: jan.pruszak@uniklinik-freiburg.de

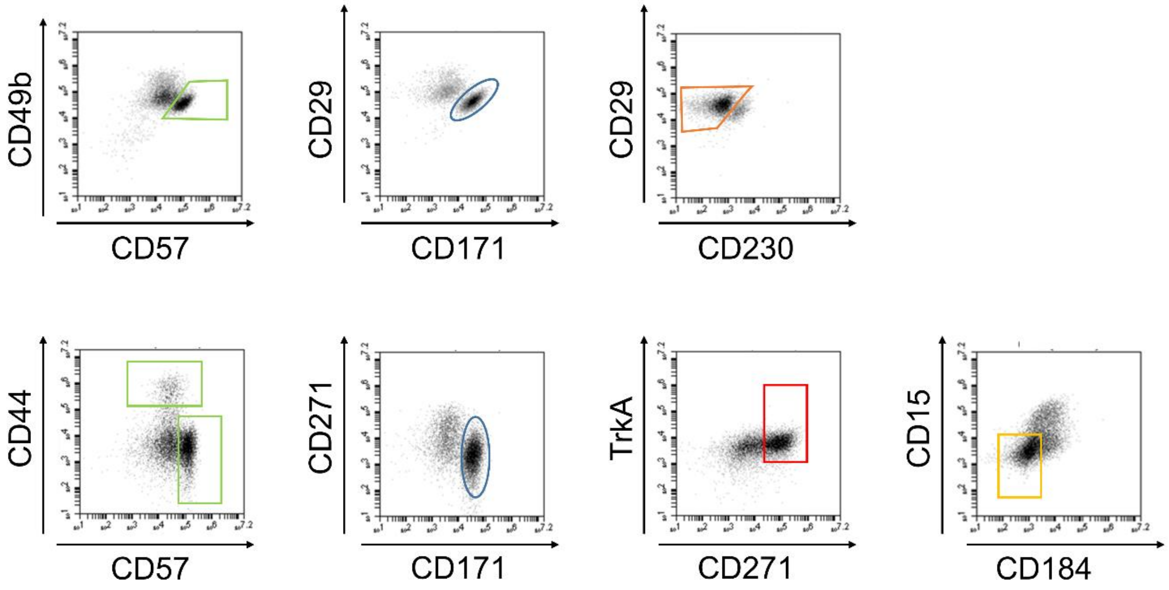

Supplementary Figure S1. Bivariate flow cytometry dot plots for further distinction of NB subpopulations. Shown are further subpopulations found in combinatorial flow cytometry analysis. Combinations with CD57 show subsets which are $CD57^{high}/CD49b^{+}$ and $CD57^{high}/CD44^{low}$. In combination with CD171, a $CD29^{high}/CD171^{high}$ and a $CD271^{low}/CD171^{high}$ subpopulation can be defined. Furthermore, a $CD29^{+}/CD230^{-}$ subpopulation can be defined. In conjunction with expression analysis and the high-throughput profiling results, the $TrkA^{+}/CD271^{high}$ and the $CD15^{-}/CD184^{-}$ subsets represent populations were deemed to be of particular relevance when aiming for *in vitro* screens to assess therapeutic responsiveness.

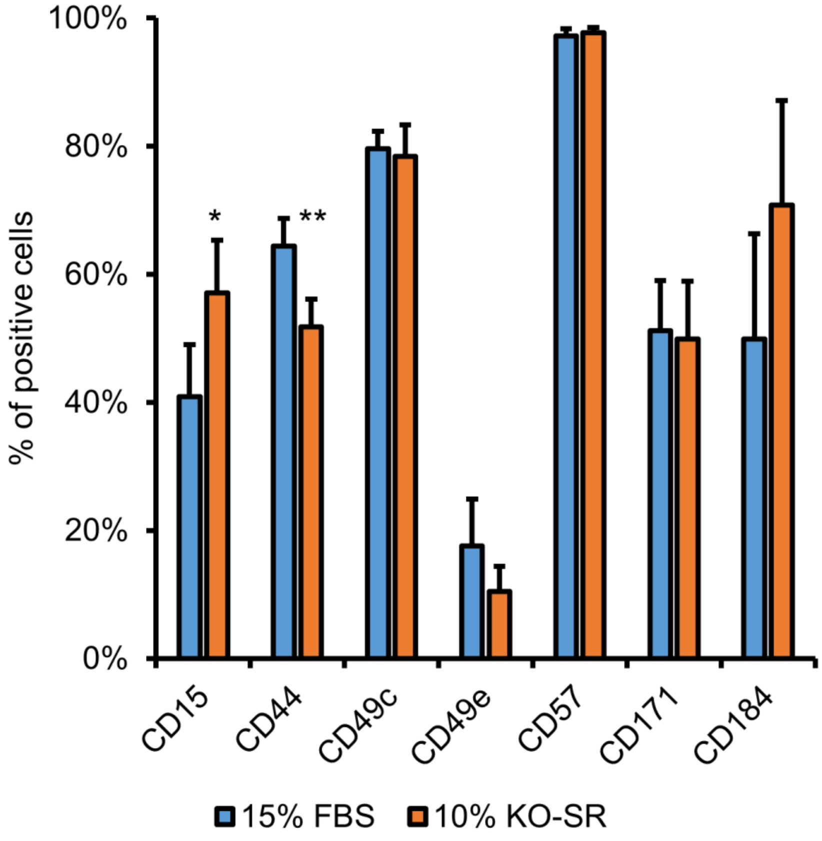

Supplementary Figure S2. Comparison of standard versus serum-free culturing conditions. Differences in baseline expression of a subset of surface antigens depending on culture medium conditions (n≥3; *p≤0.05; **p≤0.01; unpaired two-tailed Student's t-test). The decrease in CD15 expression, a marker associated with neural stemness [Pruszak *et al.*, Stem Cells 2009], might be attributed to serum components promoting neural differentiation. The increase in CD44 is in keeping with reports on serum components modulating YAP via GPCR signaling [Yu *et al.*, Cell 2012] (YAP previously identified by us to be associated with CD44 expression [Hindley *et al.*, Scientific Reports 2016]).

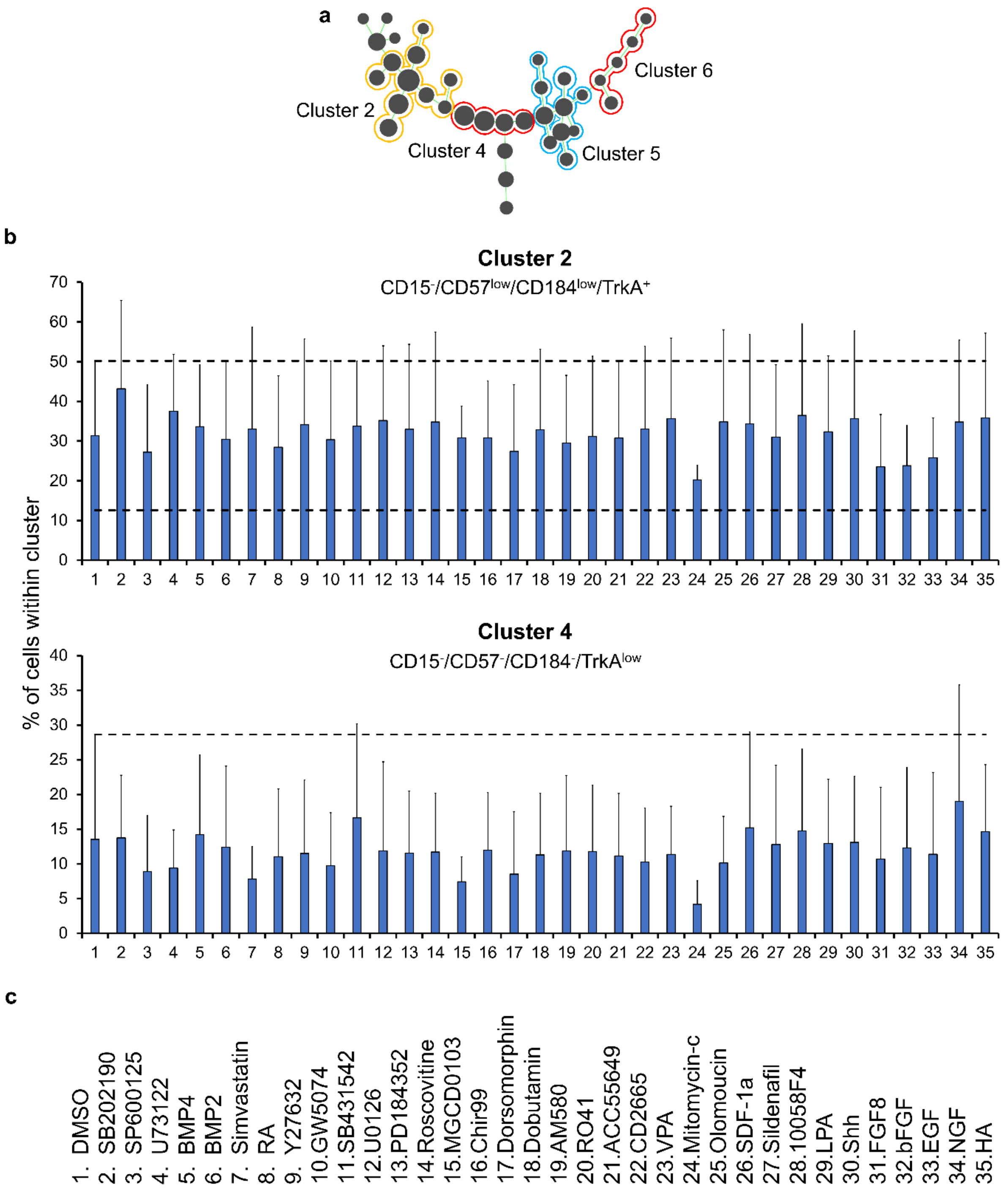

Supplementary Figure S3. Additional SPADE clusters analyzed in the small molecule screen. **(a)** SPADE clustering tree (see **Figure 2**). **(b)** No major changes were observed in clusters 2 and 4. Error bars represent standard deviation of the mean. Dashed lines represent standard deviations from DMSO control group. n≥3. **(c)** List of small molecules applied in the screen.

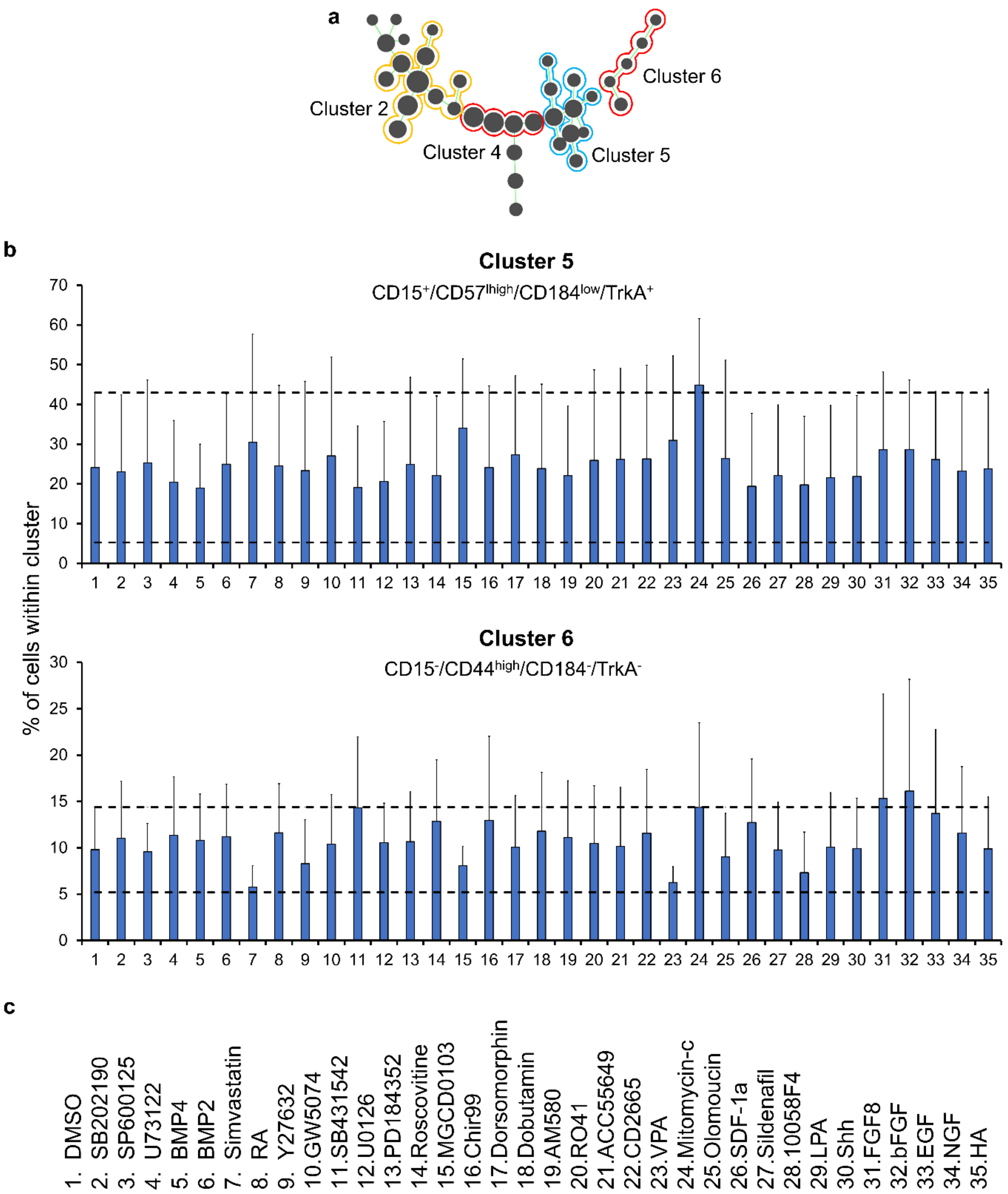

Supplementary Figure S4. Additional SPADE clusters analyzed in the small molecule screen. **(a)** SPADE clustering tree (see **Figure 2**). **(b)** No major changes were observed in clusters 5 and 6. Error bars represent standard deviation of the mean. Dashed lines represent standard deviations from DMSO control group. n≥3. **(c)** List of small molecules applied in the screen.

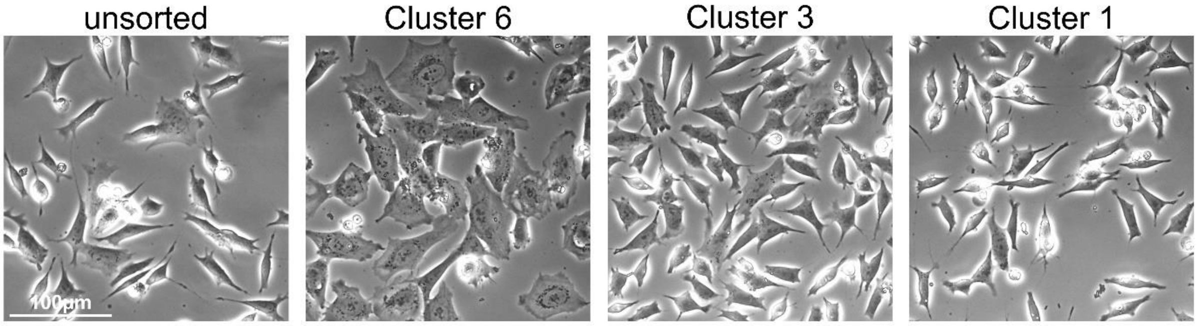

Supplementary Figure S5. Phase contrast images of SH-SY5Y cells sorted on the basis of $CD15^-/CD44^+/TrkA^-$ (cluster 6), $CD15^-/CD44^-/TrkA^+$ (cluster 3) and $CD15^+/CD44^-/TrkA^-$ (cluster 1). Morphologically, the already described CD44+ subpopulation (cluster 6) differs drastically from the other clusters (see Hindely et al., 2016). However, despite their clearly distinct responsiveness to BMP4 treatment (see **Figure 2**), no overt morphological differences were discernible between cluster 1 versus cluster**.** Scale bar =100µm.

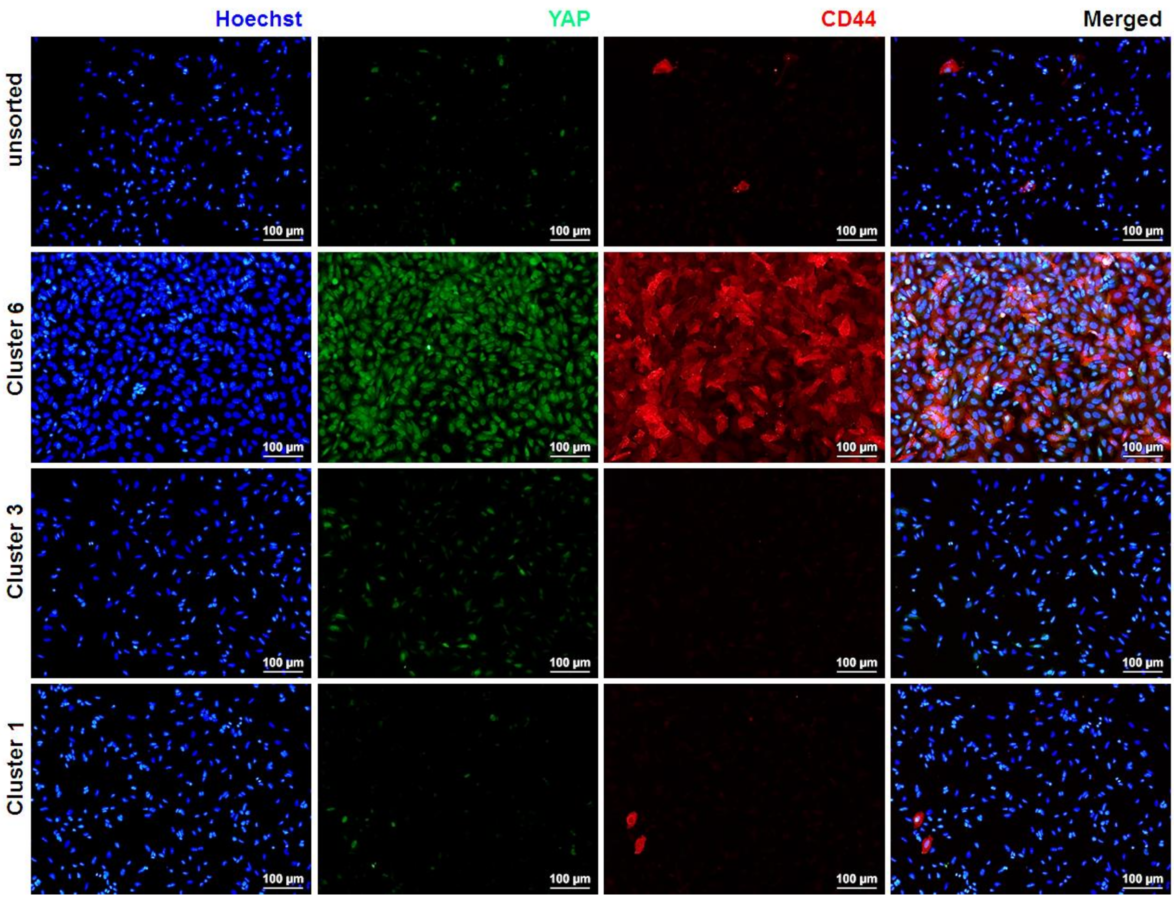

Supplementary Figure S6. Immunocytochemical analysis of SH-SY5Y subpopulations after FACS. Cluster 6 isolated on the basis of $CD15^-/CD44^+/TrkA^-$ was highly positive for YAP and CD44, whereas the $CD15^-/CD44^-/TrkA^+$ (cluster 3) and $CD15^+/CD44^-/TrkA^-$ (cluster 1) subpopulations showed nearly no expression of either marker and no striking differences between the two. Scale bar = 100µm.

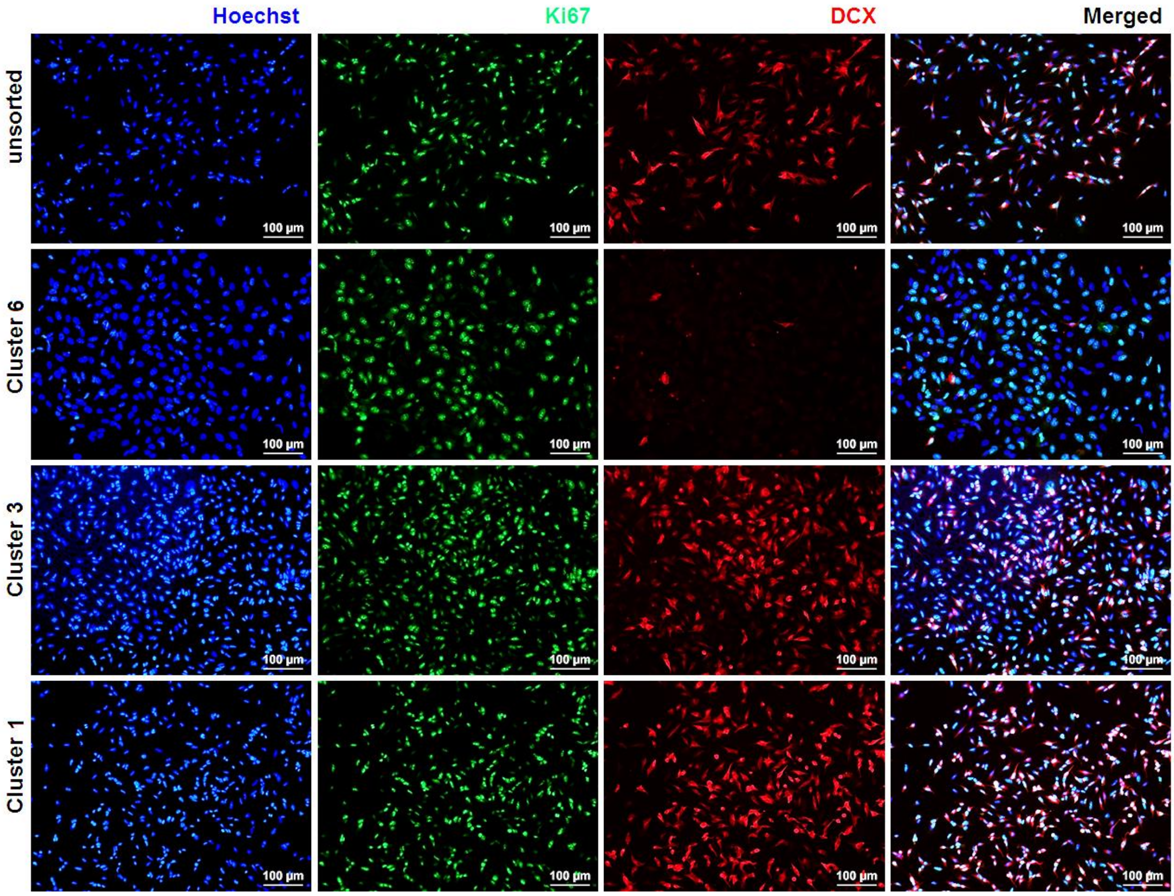

Supplementary Figure S7. Immunocytochemical analysis of SH-SY5Y subpopulations after FACS. Cluster 6 isolated on the basis of $CD15^-/CD44^+/TrkA^-$ surface expression showed nearly no immunoreactivity for DCX yet maintained expression of Ki67. In the $CD15^-/CD44^-/TrkA^+$ (cluster 3) and $CD15^+/CD44^-/TrkA^-$ (cluster 1) subpopulations, DCX and Ki67 were found to be expressed to a similar degree. Scale bar = 100µm.

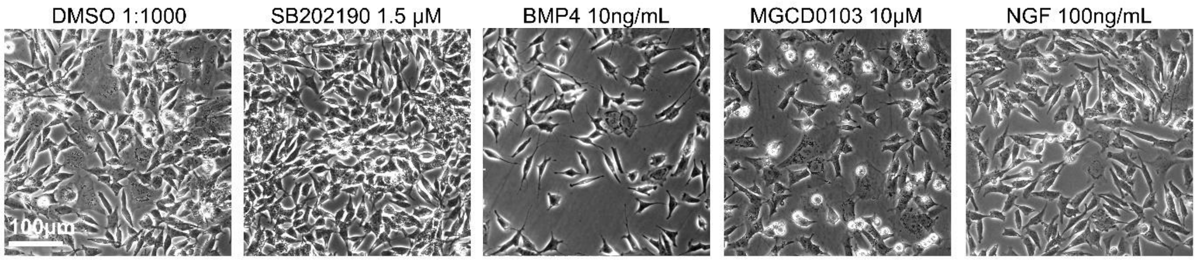

Supplementary Figure S8. Morphological changes upon small molecule treatment which showed changes in the surface marker expression patterns. Upon BMP4 treatment, neurite-like extensions were seen. Scale bar = 100µm.

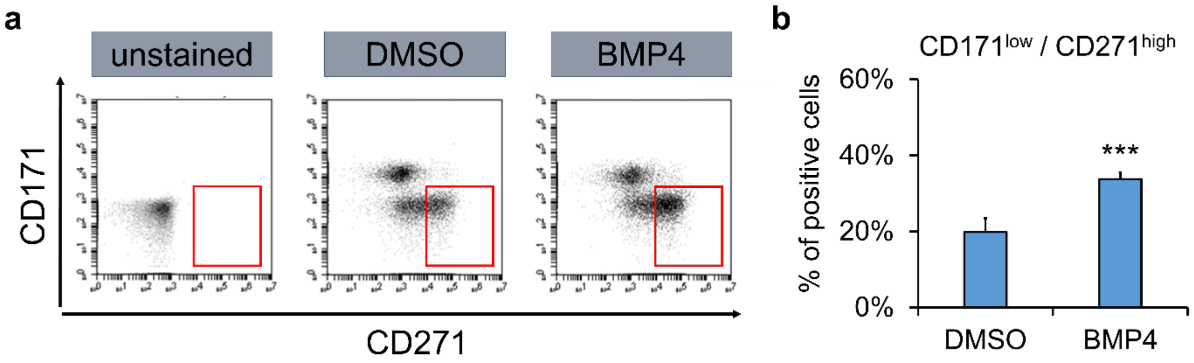

Supplementary Figure S9. Effects of BMP4 on CD171/CD271 combinatorial surface antigen expression. Treatment with BMP4 at a concentration of 10ng/mL over the course of three days increased the CD171^{low}/CD271^{high} subpopulation. n≥3; ***p≤0.001 unpaired two-tailed Student's t-test.

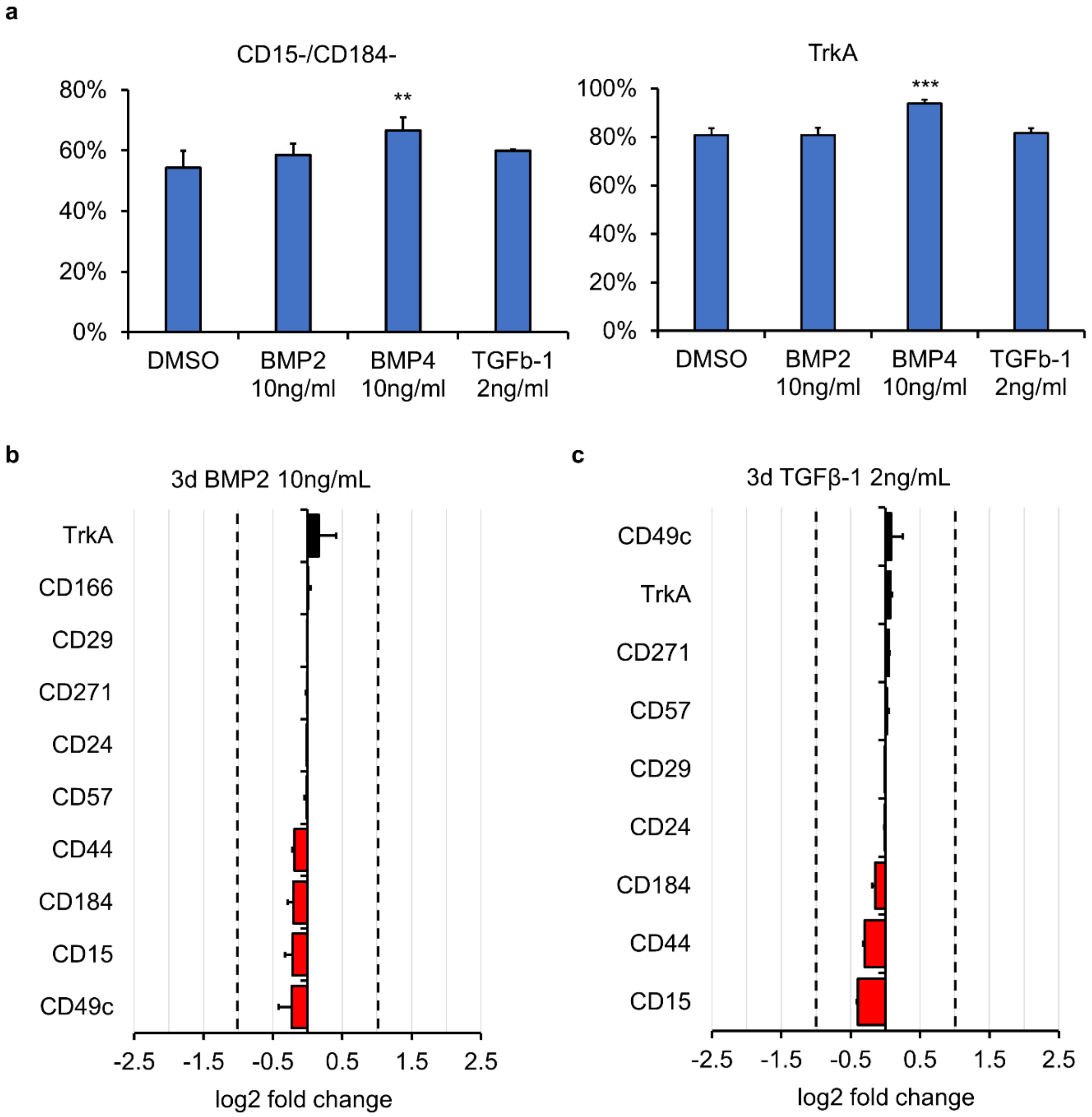

Supplementary Figure S10. (a) Quantification of flow cytometric data. Compared to DMSO control, only BMP4 was able to promote negativity for CD15 and CD184, while enhancing the expression of TrkA. n≥3, **p≤0.01, ***p≤0.001. **(b)** and **(c)** show the effects of BMP2 and TGFβ-1 on surface marker expression. Bar graphs represent log2 fold change in the expression pattern of different surface antigens after 3day 10ng/mL treatment with BMP2. n≥3; dashed lines represent cutoff of 1; red bars represent down-, black bars upregulation of the indicated surface antigens; error bars represent standard error of the mean incl. error propagation.

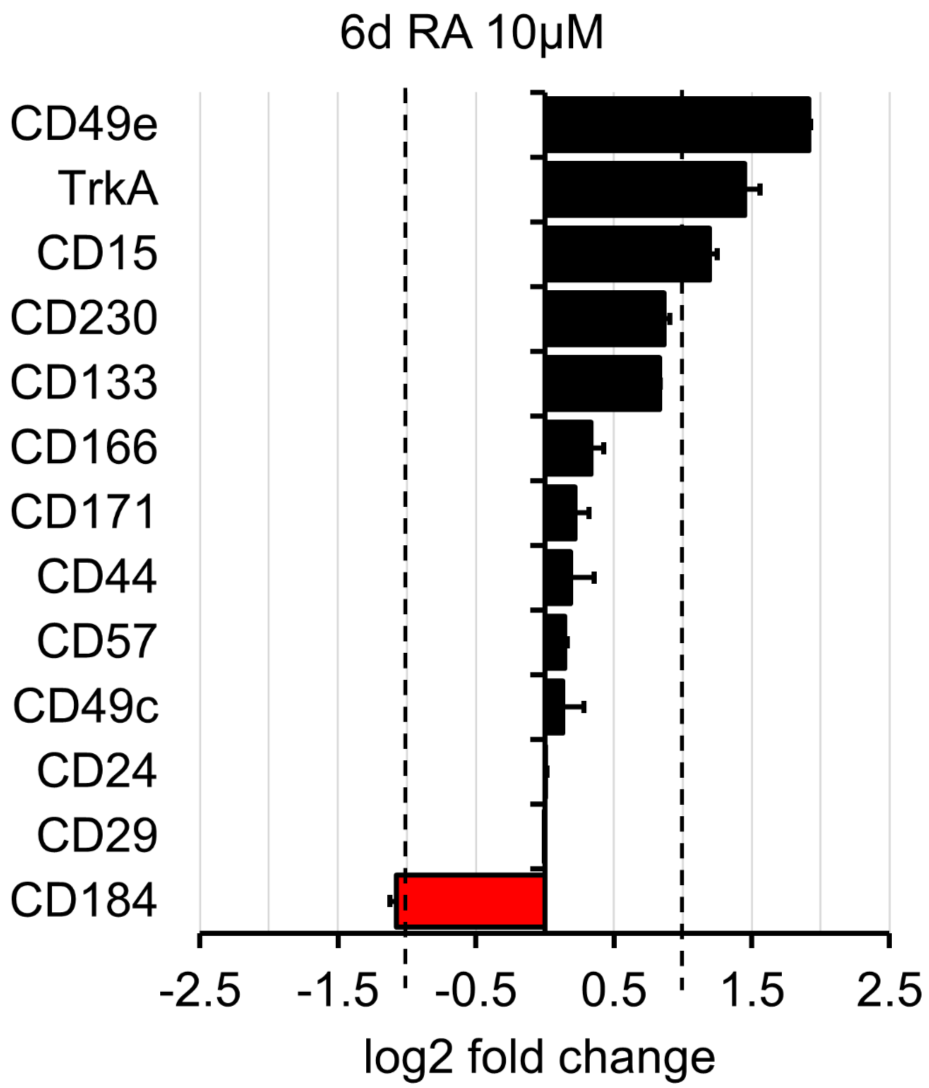

Supplementary Figure S11. Effects of RA on surface marker expression pattern. Six days of treatment with 10µM all-trans RA led to an enhancement in TrkA expression and a decrease of CD184 expression, similar to treatment with BMP4 albeit to a lesser degree. However, the expression of CD15 was enhanced upon treatment with RA. Bar graphs represent log2 fold change in the expression pattern of different surface antigens after 6day 10µM treatment with all-trans RA. n≥3; dashed lines represent cutoff of 1; red bars represent down-, black bars upregulation of the indicated surface antigens; error bars represent standard error of the mean incl. error propagation.

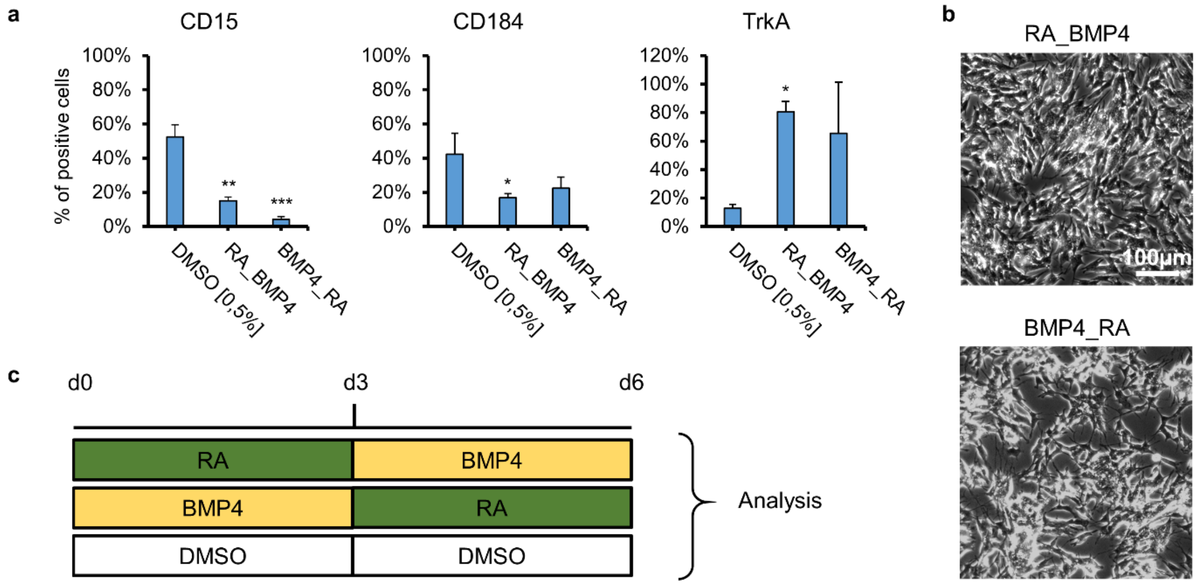

Supplementary Figure S12. For consecutive treatment with BMP4 and RA, the sequence of applying the respective treatment might be of importance. **(a)** Reduction of CD15 expression was higher, when cells were first treated with BMP4, whereas prior treatment with RA more prominently affected CD184 and TrkA expression. n≥3; *p≤0.05, **p≤0.01, ***p≤0.001 in a one-way ANOVA followed by Bonferroni's multiple comparison test. **(b)** Furthermore, the morphological changes observed after the treatments were found to differ depending on the order in which RA and BMP4 were applied. **(c)** Schematic of the treatment paradigms applied.

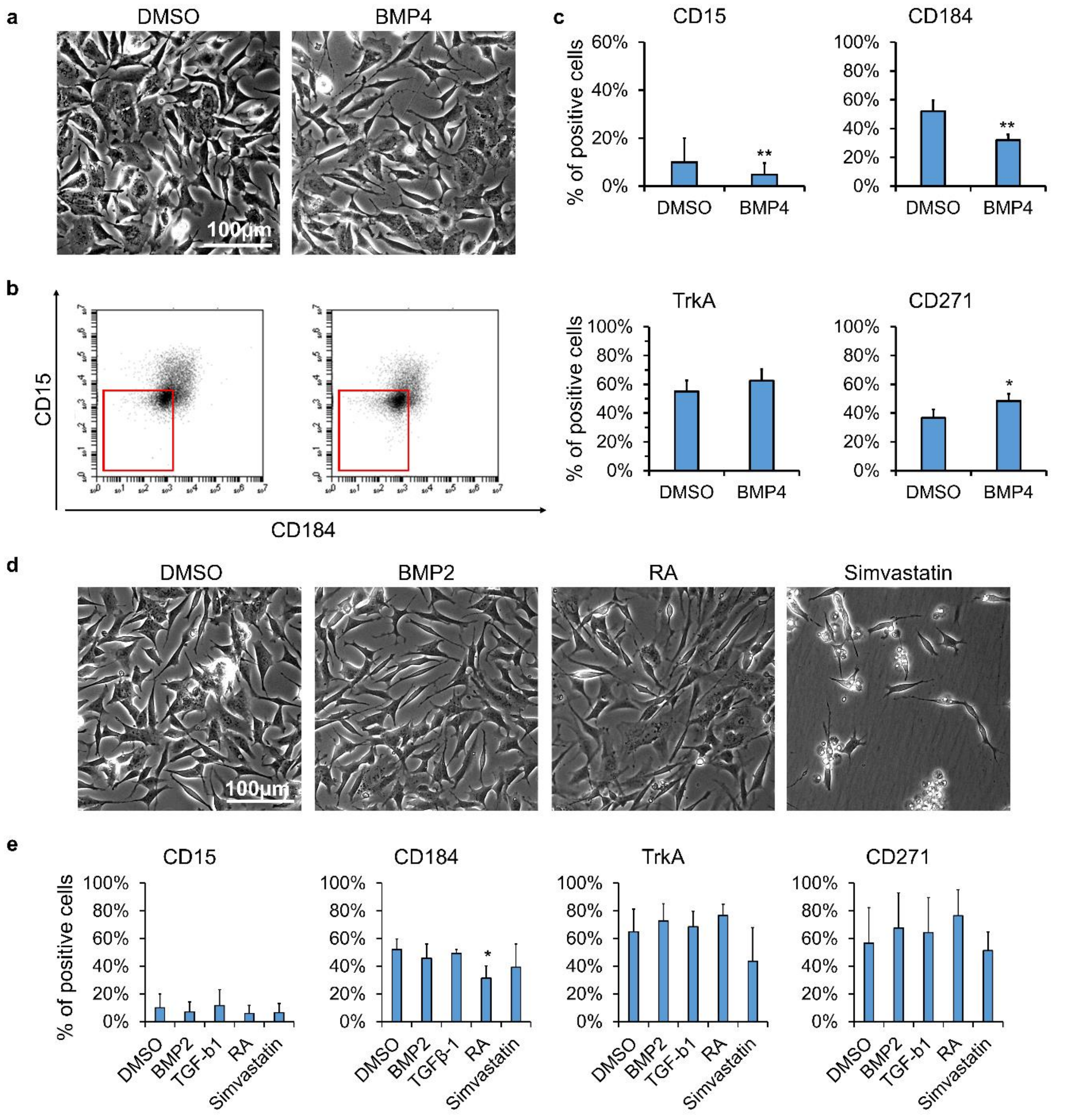

Supplementary Figure S13. Effects of a subset of small molecules in 15% FBS medium. SH-SY5Y cells were treated for 3 days with the above named small molecules. **(a)** Morphological changes with neurite-like outgrowths were observed after BMP4 treatment. **(b)** Bivariate plots showing the increase of the $CD15^-/CD184^-$ double-negative subpopulation following 3day 10ng/mL BMP4 treatment. **(c)** Quantification of flow cytometric data. n≥3; *p≤0.05, **p≤0.01 in an unpaired two-tailed Student's t-test. **(d)** Phase contrast images of the cells after 3day treatment with 10ng/mL BMP2, 10µM RA and 10µM Simvastatin, a known promoter of BMP expression. **(e)** Quantification of flow cytometry data. n≥3, *p≤0.05, in a one-way ANOVA followed by Dunnett's multiple testing comparing all values with the DMSO control.

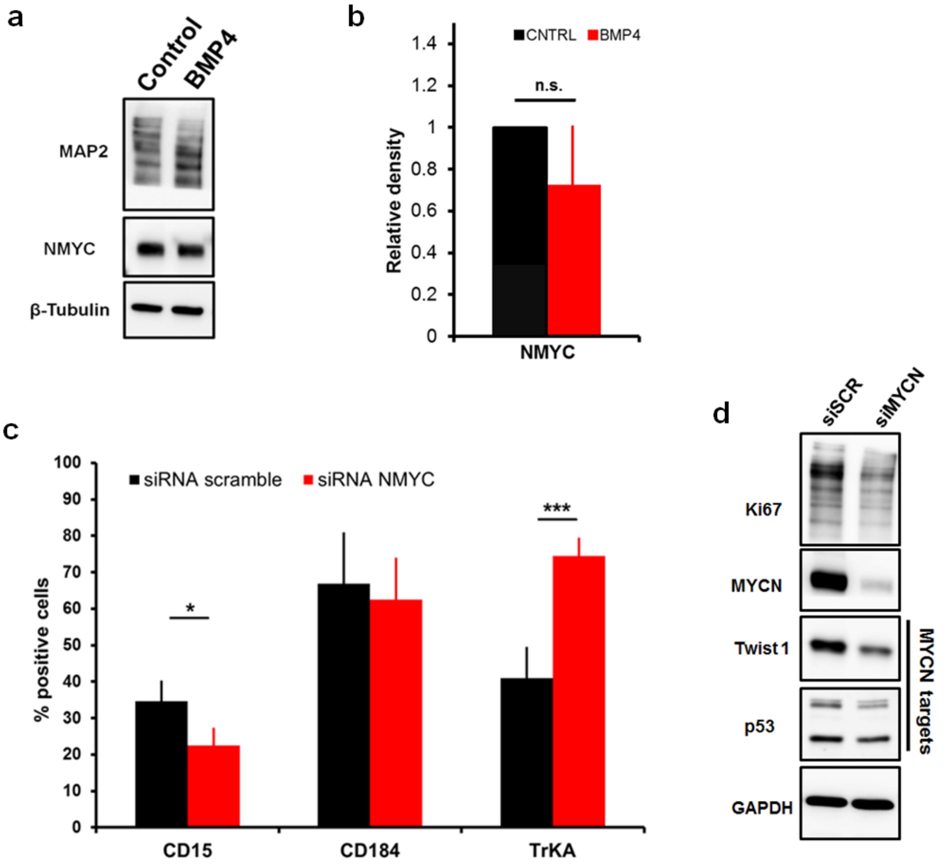

Supplementary Figure S14. BMP4 treatment and siRNA-mediated knockdown of *NMYC* in BE(2)-M17 cells. **(a)** MAP2 showed a similar trend towards enhancement after BMP4 treatment as Synapsin without reaching significance (n=3; see **Figure 5 d**). **(b)** In contrast to SH-SY5Y cells (see **Figure 5 e**), no overt changes on NMYC protein content were discernible in BE(2)-M17 cells after BMP4 treatment when applying the same regimen (10 ng/mL over 3 days). **(c)** After 2 days of siRNA treatment (*NMYC* vs. scramble control), surface marker changes and protein content were assessed. *NMYC* knockdown resulted in decreased CD15 and increased TrkA surface expression (n=4). **(d)** Immunoblot analysis of *NMYC* knockdown by siRNA and resulting effect on proliferative marker ki-67 and NMYC targets.

Supplementary Materials and Methods

Supplementary Table S1 Antibodies used for the small molecule screen

Antibody	Conjugated	Clone	Company	Reference #	Dilution
**CD 15**	FITC	H198	eBioscience®	11-0159-42	1:50
**CD 24**	eFluor780	eBio SN3	eBioscience®	47-0247	1:50
**CD 44**	APC	IM7	eBioscience®	17-0441-83	1:50
**CD 57**	eFluor460	TB01	eBioscience®	48-0577	1:50
**CD 184**	PerCP-Cy5.5	12G5	BD Pharmingen™	560670	1:50
**TrkA**	PE	REA430	Miltenyi Biotec	130-106-606	1:50

Supplementary Table S2 Small molecules

Molecule	Concentration	Company	Reference #
**10058-F4**	50 µM	Santa Cruz	Sc-213577
**ACC55649**	100 nM	Tocris bioscience	2436
**AM580**	500 nM	Santa Cruz	Sc-203505
**bFGF**	20 ng/mL	PreproTech	100-18C
**BMP2**	10 ng/mL; 100ng/mL	PreproTech	120-02
**BMP4**	10 ng/ml; 100ng/mL	PreproTech	120-05ET
**CD2665**	100 nM	Santa Cruz	Sc-293988
**Chir99**	6 µM	Tocris bioscience	4423
**Dobutamin**	10 µM	Sigma	D0676
**Dorsomorphin**	1 µM	Stemgent	04-0024
**EGF**	20 ng/mL	PreproTech	AF-100-15
**FGF8**	100 µg/mL	PreproTech	100-25
**GW5074**	1 µM	Sigma-Aldrich	G6416
**Hyaluronic Acid**	1 mg/mL	R&D Systems	GLR003
**LPA**	5 µM	Santa Cruz	Sc-201053
**MGCD0103**	10 µM	Sellenckchem	S1122
**Mitomycin-c**	3 µM	Sigma Life Science	M0503
**NGF**	100 ng/mL	PreproTech	450-01
**Olumoucine**	10 µM	Sigma Life Science	O08864
**PD184352**	1 µM	Sigma	PZ0181
**Retinoic Acid (all-trans)**	10 µM	Sigma	R2625
**RO41-5253**	50 nM	Santa Cruz	Sc-471345
**Roscovitine**	2.5 µM	Sigma Life Science	R7772
**SB202190**	1.5 µM	Sellenckchem	1077
**SB431542**	10 µM	Miltenyi Biotec	130-097-448
**SDF-1α**	20 nM	PreproTech	300-28A
**Shh**	100 ng/mL	R&D Systems	1314-SH
**Sildenafil**	10 µM	Sigma	PZ0003
**Simvastatin**	10 µM	Sigma	S6196
**SP600125**	10 µM	Sellenckchem	S1460
**TGF-β1**	2 ng/mL	PreproTech	100-21
**U0126**	5 µM	Sigma Aldrich	U120
**U73122**	2 µM	Tocris	1268
**VPA**	2 mM	Sigma	P4543
**Y27632**	10 µM	Selleckchem	S1049

Supplementary Table S3 Antibodies used for flow cytometry

Conjugated	Antibody	Clone	Company	Reference #	Dilution
**FITC**	CD15	H198	eBioscience®	11-0159-42	1:50
	CD24	eBioSN3	eBioscience®	11-0247-42	1:50
	CD29	TS2/16	eBioscience®	11-0299-42	1:50
	CD49b	eBioY418	eBioscience®	11-0498-42	1:50
	CD49e	eBioSAM-1	eBioscience®	53-0469-42	1:50
	CD57	TBO1	eBioscience®	11-0577-42	1:50
**AlexaFluor® 488**	CD166	3A6	AbD Serotec®	MCA 1926A488	1:50
**PE**	CD29	TS2/16	eBioscience®	12-0299-42	1:50
	CD49d	9F10	eBioscience®	12-0499-42	1:50
	CD57	TB01	eBioscience®	12-0577-42	1:50
	CD171	eBio5G3	eBioscience®	12-1719-42	1:50
	CD184	12G5	eBioscience®	12-9999-42	1:50
	CD230	4D5	eBioscience®	12-9230-42	1:50
	TrkA	REA430	Miltenyi Biotec	130-106-606	1:50
**APC**	CD29	TS2/16	eBioscience®	17-0299-42	1:50
	CD44	IM7	eBioscience®	17-0441-83	1:50
	CD49c	P1B5	eBioscience®	17-0494-42	1:500
	CD133	AC1/133	Miltenyi Biotec	130-090-826	1:50
	CD184	12G5	eBioscience®	17-9999-42	1:50
**AlexaFluor® 647**	CD271	C40-1457	BDBiosciences	560326	1:50

Supplementary Table S4 Antibodies used for immunocytochemistry

Antibody	Conjugated	Clone	Company	Reference #	Dilution
Ki67	Rabbit	MM1	Novocastra	NCL-Ki67p	1:1000
DCX	Goat	C-18	Santa Cruz	Sc-8066	1:200
TUJ1	Mouse	TUJ1	Covance	MMS-435P	1:1000
NCAM	Mouse	F-4	Santa Cruz	Sc-106	1:200
SOX2	Goat		R&D Systems	AF2018	1:500
YAP1	Rabbit	H-125	Santa Cruz	Sc-15407	1:100
CD24	Mouse	SN3	Santa Cruz	Sc-19585	1:100
CD29-APC	Mouse	TS2/16	eBioscience®	17-0299-42	1:50
CD44-APC	Rat	IM7	eBioscience®	17-0441-83	1:50
CD49c-APC	Mouse	PIB5	eBioscience®	17-0494-42	1:50
CD57	Mouse	HNK-1	BDPharmingen®	559048	1:100
CD15-FITC	Mouse	H198	eBioscience®	11-0159-42	1:50
CD133-APC	Mouse	AC1/133	Miltenyi Biotec	130-090-826	1:50
CD171-PE	Mouse	eBio5G3	eBioscience®	12-1719-42	1:50
CD271-AF647®	Mouse	C40-1457	BDBiosciences	560326	1:50

Supplementary Table S5 Buffers and other products for western blot

Buffer	Composition	Company	Reference #
**Sample buffer**	0.4 M Tris-HCl (pH6.8)<br>6% SDS<br>30% Glycerol	Carl Roth®<br>SERVA<br>Carl Roth®	9090.3<br><br>3783.1
**Running buffer**	25mM Tris base<br>192mM Glycine<br>1% SDS	AppliChem<br>AppliChem<br>SERVA	A1379<br>A1377
**Transfer buffer**	25mM Tris base<br>192mM Glycine<br>20% methanol	AppliChem<br>AppliChem	A1379<br>A1377
**Blocking buffer**	4% milk powder in PBST	Sigma-Aldrich®	70166
**PBS**	9.54g PBS buffer powder<br>H_2O	AppliChem	A0965,9010
**PBST**	0.2% Tween in 1x PBS	AppliChem	A1389

Supplementary Table S6 Antibodies for western blot

Antibody	Host	Company	Reference #	Dilution
GAPDH (ID4)	Mouse	Santa Cruz	Sc-59540	1:5000
β-Tubulin	Rabbit	Abcam	Ab6046	-
NMYC	Mouse	Santa Cruz	Sc-53993	1:500
DCX	Goat	Santa Cruz	Sc-8066	1:500
MAP2	Mouse	Millipore	MAB3418	1:200
Synapsin	Rabbit	Sigma	S193	1:500
Precision plus ProteinTM WesternCTM Standard		BioRad	161-0376	1:10000

Supplementary Table S7 RNA interference

Name	Supplier	Catalog No	Final conc.	Sequence
siMYCN_1	Origene	SR303027A	50nM	CGCUGAUACAU AACUAAAUUUG ATA
siMYCN_2	Origene	SR303027B	50nM	AGUUCAUACCU AAGUACUGUAA UAA
siMYCN_3	Origene	SR303027C	50nM	AGCUGAUCCUC AAACGAUGCCU UCC
siSCRL	Origene	SR30004	50nM	CGUUAAUCGCG UAUAAUACGCG UAT

References

Hindley, C. J. *et al.* The Hippo pathway member YAP enhances human neural crest cell fate and migration. *Sci. Rep.* **6,** 23208 (2016).

Pruszak, J. *et al.* CD15, CD24, and CD29 define a surface biomarker code for neural lineage differentiation of stem cells. *Stem Cells*. **27**, 2928-40 (2009).

Yu, F.X. *et al.* Regulation of the Hippo-YAP pathway by G-protein-coupled receptor signaling. *Cell*. **150**, 780-91 (2012).
